# Revealing the Role of Noncovalent Interactions on the Conformation of the Methyl Group in Tricyclic Orthoamide

**DOI:** 10.1021/acs.joc.3c02016

**Published:** 2023-12-15

**Authors:** Jorge Gutiérrez-Flores, Eduardo H Huerta, Gabriel Cuevas, Jorge Garza, Rubicelia Vargas

**Affiliations:** † Departamento de Química, División de Ciencias Básicas e Ingeniería, Universidad Autónoma Metropolitana Iztapalapa, San Rafael Atlixco 186, Col. Vicentina, Iztapalapa, C.P. 09340 Ciudad de México, México; ‡ Insituto de Química, Universidad Nacional Autónoma de México, Circuito Exterior, Ciudad Universitaria, Alcaldía Coyoacán C.P. 04510 Ciudad de México, México

## Abstract

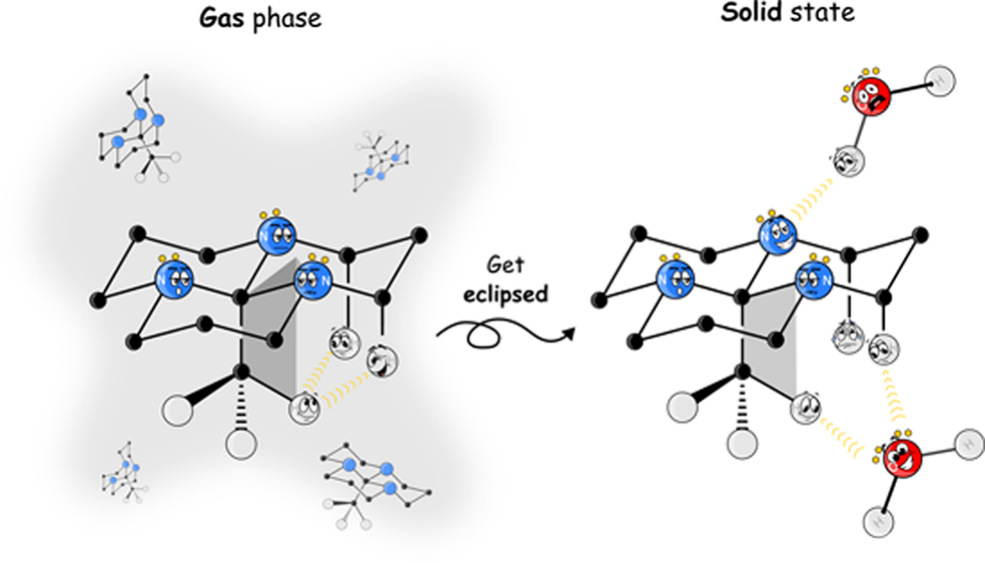

Tricyclic orthoamides are valuable molecules with wide-ranging applications, including organic synthesis and molecular recognition. Their structural properties make them intriguing, particularly the eclipsed all-*trans* conformer, which is typically less stable than the alternated conformation and is a rare phenomenon in organic chemistry. However, it gains stability in crystalline and hydrated settings, challenging the existing theoretical explanations. This study investigates which factors make eclipsed conformers more stable using experimentally reported anhydrous (ATO) and hydrated (HTO) crystal structures. Employing the quantum theory of atoms in molecules, noncovalent interaction index, and pairwise energy decomposition analysis, we delve into the noncovalent interaction environment surrounding the molecule of interest. In ATO, dispersive interactions dominate, whereas in HTO, both dispersive and electrostatic contributions are observed due to the presence of water molecules. Anchored to the lone pairs of the nitrogen atom in the orthoamide tricycle, water molecules prompt the methyl group’s eclipsing through intermolecular and intramolecular interactions. This work resolves the long-standing conflict behind why tricyclic orthoamide has an eclipsed conformation by establishing the stabilization factors. These insights have implications for crystal engineering and design, enhancing our understanding of structural behavior in both crystalline and hydrated environments.

## 1 Introduction

**Figure 1:**
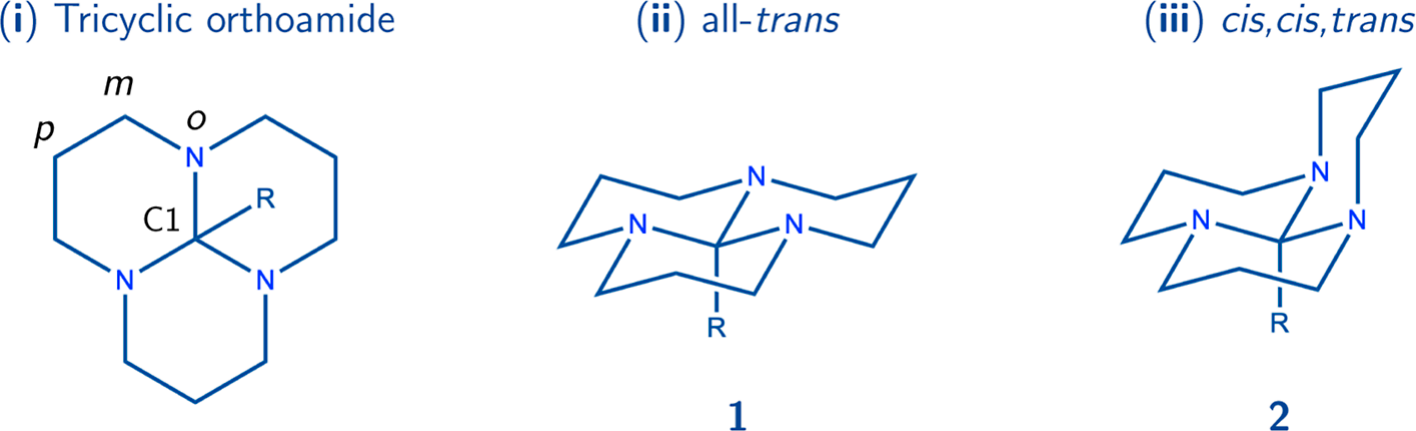
Structural representation of tricyclic orthoamide, where a: R = H and b: R = CH_3_. (i) Linear representation: identifying ortho (*o*), meta (*m*), and para (*p*) positions. (ii) All-*trans* configuration and (iii) *cis,cis,trans* configuration.

*Tricyclic orthoamides* [Figure 1(i)] constitute a significant class of organic compounds due to their versatile characteristics. These compounds have demonstrated the ability to serve as organic structures for hydride ion donation, making them valuable in chemical reactions involving reduction processes.^1,2^ Additionally, they function as Lewis bases, providing multiple interaction points for coordination with metal ions and other electron-deficient species.^3,4^ Hence, these compounds are valuable building blocks in constructing chelator macrocyclic molecules, which find applications in metal-ion sensing and extraction.^5−7^ Due to the remarkable attributes exhibited by tricyclic orthoamides, they have garnered considerable interest, making them the subject of study in various fields, including organic synthesis, materials science, and supramolecular and macromolecular chemistry.

The notable structural architecture exhibited by tricyclic orthoamides is the key factor behind the emergence of their exceptional and unusual properties. Tricyclic orthoamide in its all-chair, all-*trans* configuration **1** [Figure 1(ii)], unveils intriguing NMR and IR spectroscopic properties in the substituents attached to the central carbon atom C1, which connects the three rings. The antiperiplanar arrangement of the three nitrogen lone pairs (LPs) and the C1–R bond rationalizes the emergence of these properties, as it promotes the *n*_N_ → σ_C1–R_* interaction making the C1–R bond different from the others of the same type.^8−10^ Furthermore, in the trihydrated crystalline form of tricyclic orthoamide **1**b, an intriguing phenomenon of eclipsing occurs within its structure. This phenomenon involves alignment between the C–H bonds of a substituted methyl group attached to carbon C1 and the three central C1–N bonds of the fused rings. X-ray diffraction analysis reveals that the dihedral angle determined by the N–C1–C–H motif, containing the mentioned bonds, is 8.0°.^11^ Moreover, the observed eclipsing of the methyl group is not an inherent property of the molecule, as indicated by adopting a typical alternate configuration, also known as staggered conformation, in the anhydrous crystal structure, which contains configurations **1**b and **2**b of the tricyclic orthoamide [Figure 1(ii,iii)].^12^ This experimental evidence, combined with theoretical studies,^13^ leads to the idea that the observed eclipsation results from directed interactions C–H···O between the hydrogens of the methyl group and the oxygen of the surrounding water molecules within the trihydrated crystalline structure.

This *eclipse phenomenon*, which involves carbon atoms with sp^3^ hybridization, is a rare occurrence among molecular systems, observed only in a limited number of cases.^14,15^ The arrangement of atoms within a molecule exhibits a preferential alternation to achieve a configuration with minimum energy, which ensures thermodynamic stability. In contrast, an eclipsed array corresponds to a higher-energy and inherently unstable structure. The origin of the increase in energy and the instability acquired when going from an alternate arrangement to an eclipsed one has been interpreted in terms of orbital interaction,^16,17^ energy analysis,^18^ forces acting on molecular electron density,^19^ and combining both dynamic orbital forces (DOF) and noncovalent interaction index (NCI).^20^ On this basis, tricyclic orthoamide is a valuable molecular prototype for exploring the mechanisms and forces that mediate conformational changes, as it adopts an unfavorable configuration under specific conditions. To date, there has been no investigation to understand the nature of the interactions occurring within the crystalline structure that play a role in stabilizing an energetically unfavorable configuration. These interactions are crucial, as they have the potential to modify the molecular potential energy surface.

This work seeks to establish a deeper understanding of the forces behind the conformational change in the tricyclic orthoamide crystal structure. To accomplish this, we conduct a rigorous investigation of the intra- and intermolecular interactions within the crystalline structures reported. We present evidence for these interactions by employing the framework of NCI and quantum theory of atoms in molecules (QTAIM), using theoretical electron density, which has demonstrated its comparability to experimental electron density.^21^ The findings not only show the remarkable chelating capacity of tricyclic orthoamides in capturing water molecules but also highlight the dominant role of weak interactions in driving the preferred eclipsing configuration. This research holds an intrinsic fascination for chemists as it aims to elucidate the involvement of noncovalent interactions in stabilizing one conformer over the others. This knowledge is a prerequisite for harnessing noncovalent interactions to control dynamic processes. By gaining a deeper understanding of these mechanisms, we can establish fundamental principles for manipulating atomic spatial arrangements, thereby influencing molecular properties such as shape, size, polarity, and linearity, impacting a wide range of physical^22−24^ and chemical processes.^25,26^

## 2 Methodology

In this study, we utilized the experimental crystallographic structures reported by Seiler and Dunitz.^12^ Our focus was on two forms of tricyclic orthoamide: the trihydrated form (HTO) and the anhydrous monoclinic form (ATO). Refer to Table S1 in the Supporting Information for the detailed crystallographic information on both ATO and HTO systems. The HTO crystal exhibits a *Pa*3 (*Z* = 8) space group and represents the eclipsed conformation of the tricyclic orthoamide. On the other hand, the ATO crystal belongs to space group *P*21/*c* (*Z* = 8) and represents the alternated (staggered) conformation. It is important to note that the ATO crystal contains two possible configurations: referred to as all-*trans* and *cis,cis,trans*. The three fused rings in both configurations are in a chair conformation. The descriptors “*cis*” and “*trans*” indicate the orientation of the nitrogen atoms’ LPs concerning the substituent on the central carbon atom. In the all-*trans* configuration, the three LPs are *anti*-periplanar-oriented (close to 180°) with respect to the C–CH_3_ bond. However, in the *cis,cis,trans* configuration, two LPs are *syn*-periplanar-oriented (close to 60°), while the third LP remains *anti*-periplanar. For more details of these descriptors, see ref (27).

Due to the limited precision of hydrogen atom positions determined by conventional X-ray techniques used in the structural determination of crystalline systems, we optimized the coordinates of the hydrogen atoms while keeping the positions of the heavy atoms and cell parameters constrained. The study of the influence of water molecules on the eclipsing of the methyl group attached to C1 followed a meticulous path. Starting within the hydrated environment of HTO, we alternated the configuration of the methyl group to obtain the HTO-A system. Afterward, we removed the water molecules from the HTO crystal, resulting in a modified tricyclic orthoamide crystal (MTO). In this modified system, we examined both the alternated conformation (MTO-A) and the eclipsed conformation (MTO-E).

The calculations were performed using the solid-state chemistry and physics CRYSTAL14 code^28^ within the density functional theory (DFT) framework.^29,30^ We employed the B3LYP exchange–correlation functional^31−34^ with Grimme dispersion correction^35^ for DFT partial geometry optimizations and the POB-DZVP(rev2)^36^ basis set for all atoms. After achieving the geometry optimizations, we conducted an analysis of the electron density using the QTAIM^37^ and NCI^38,39^ tools implemented in the GPUAM code.^40,41^ This allowed us to capture all the noncovalent interactions present in the systems.

The QTAIM analysis entails a topological examination of electron density ρ(**r**) by looking for critical points (CPs) where ∇ρ(**r**) = 0. These CPs are classified based on range (ω) and curvature (σ), which depend on the second derivative of ρ(**r**) at CP positions. They are categorized as nuclear (NCP), bond (BCP), ring (RCP), and cage (CBP) CPs, corresponding to (3,3), (3, −1), (3, +1), and (3, +3) (ω, σ) pairs, respectively. Usually, BCPs are present between interacting atoms^42^ which are connected through bond paths (BPs), but they do not provide information about the nature of the interaction (covalent or noncovalent). Complementary tools like NCI are employed to classify and identify specific noncovalent interactions. NCI utilizes the adimensional index *s*(**r**), the reduced gradient density, which depends on ρ(**r**) and its gradient ∇ρ(**r**). It is expressed as .^38^ To discern the type of weak interaction, NCI employs the density Laplacian ∇^2^ρ(**r**), which satisfies the relation ∇^2^ρ(**r**) = λ_1_ + λ_2_ + λ_3_, where λ_*i*_ represents Hessian’s eigenvalues.^37,42^ NCI’s model utilizes the second eigenvalue’s sign as an indicator of bonding [sign(λ_2_) < 0] or nonbonding [sign(λ_2_) > 0] interactions. The behavior of *s*(**r**) against sign(λ_2_)·ρ(**r**) is depicted in an NCI plot, distinguishing attractive interactions [sign(λ_2_)·ρ(**r**) < 0], weakly attractive interactions [sign(λ_2_)·ρ(**r**) ≈ 0], and nonattractive interactions [sign(λ_2_)·ρ(**r**) > 0].^38^ These NCI surfaces generally align with CPs in the density gradient, particularly for directional interactions.^43^

To deepen our understanding of noncovalent interactions, we performed a pairwise energy decomposition analysis (EDA) using an all-*trans* orthoamide as a reference molecule in both the ATO and HTO crystals. The EDA involved calculating the interaction energies for a cluster of molecules located within a 5.0 Å radius around the center of mass of the reference molecule. This analysis was carried out using the CrystalExplorer software,^44^ at B3LYP/6-31G(d,p)^45−47^ level of theory, allowing us to gain valuable insights into the nature and contributions of noncovalent interactions within the crystal structures.

## 3 Results and Discussion

### 3.1 Geometry and Energy Analysis

**Figure 2:**
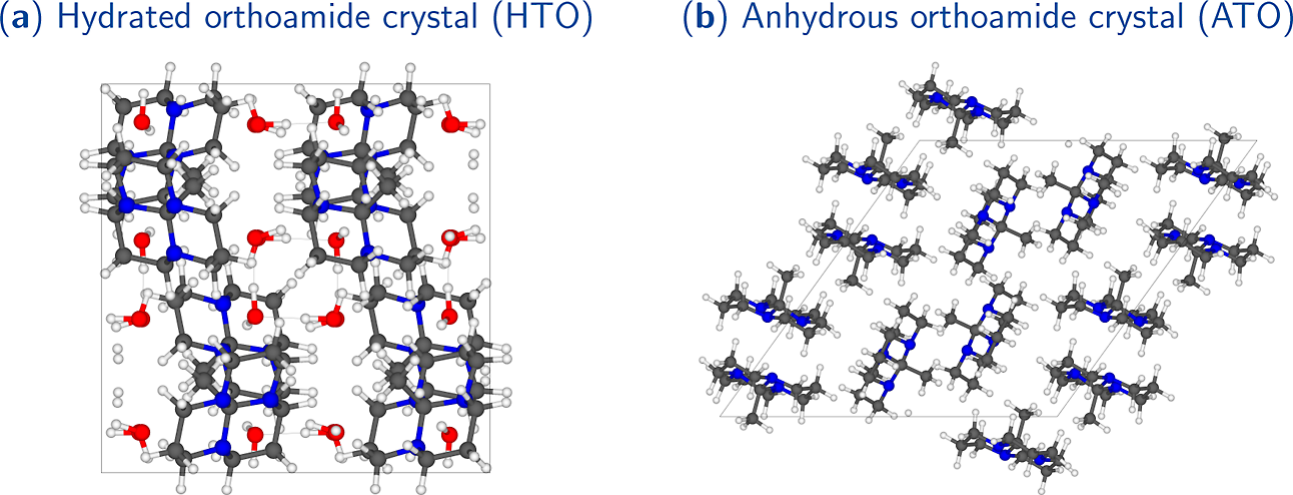
Optimized crystal structures. Only the relaxation of the hydrogen atoms was allowed. The positions of the heavy atoms (C, N, and O) and the cell parameters were fixed. The fractional coordinates and cell parameters are reported in the Supporting Information.

**Figure 3:**
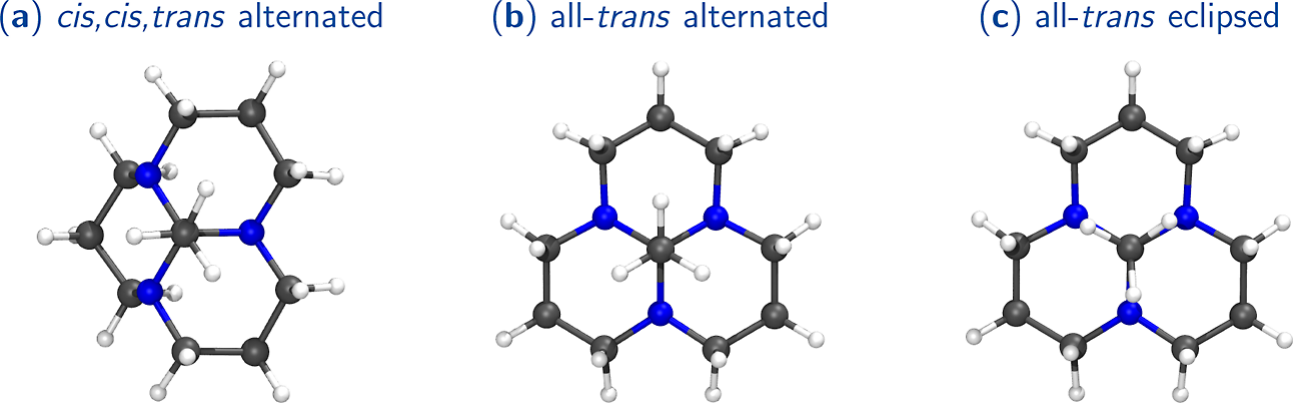
Orthoamide’s alternate and eclipsed conformers. *Cis,cis,trans* (a) and all-*trans* (b) alternated configurations were obtained from the ATO crystal, whereas the all-*trans* eclipsed configuration (c) was extracted from the HTO crystal.

Figure 2 depicts the optimized structures of the hydrated (HTO) and anhydride (ATO) tricyclic orthoamide crystals. In the HTO crystal, the orthoamide exclusively adopts the all-*trans* configuration (Figure 3c), while the methyl group attached to the central carbon atom is nearly eclipsed [mean dihedral angle, ϕ̅(N–C1–C–H), equal to 5.25°]. In contrast, the ATO crystal exhibits the coexistence of both all-*trans* (Figure 3b) and *cis,cis,trans* (Figure 3a) configurations. Notably, regardless of the configuration, the methyl group in both cases remains alternated .

The presence of water molecules constitutes the primary distinction among these systems. Therefore, based on their geometric properties, it can be inferred that water molecules play two crucial roles: (a) determining the preference for the all-*trans* configuration and (b) stabilizing the eclipsed conformation of the methyl group.

First, to investigate the coexistence of the all-*trans* (Figure 3b) and *cis,cis,trans* (Figure 3a) structures in the anhydride crystal, we conducted a comprehensive theoretical thermodynamic study, under standard-state conditions, in both the gas phase and solution. For the latter, a continuous solvation model was used considering water and acetone as solvents. Please refer to the Supporting Information for detailed information and results. Our thermodynamic analysis revealed that at equilibrium, both configurations could be present, as indicated by low values of Δ_inv_*G*° (inversion Gibbs free energy) and *K* (equilibrium constant). Specifically, in the gas phase, water, and acetone, the Δ_inv_*G*° values are −0.12, 1.70, and 1.07 kcal/mol, while the corresponding *K* values are 1.224, 0.057, and 0.164, respectively. Consequently, in an anhydrous crystalline environment, the presence of both all-*trans* and *cis,cis,trans* configurations is expected (Figure 2b). However, the equilibrium seems to shift toward the all-*trans* configuration in the presence of water molecules within the molecular crystal (Figure 2a). Therefore, water molecules play a critical role in conferring a preference to the all-*trans* conformation. Figure 2a visually demonstrates a cluster of water molecules located in the central region of the HTO unit cell. These water molecules engage in hydrogen bonding interactions with the LPs of the orthoamide’s nitrogen atoms (*r*_N···H_ = 1.91 Å and *∠*_O–H···N_ = 162.38°, further elaborated in subsequent discussions). These interactions primarily account for the prevalent existence of the all-*trans* structure in the HTO crystal.

**Figure 4:**
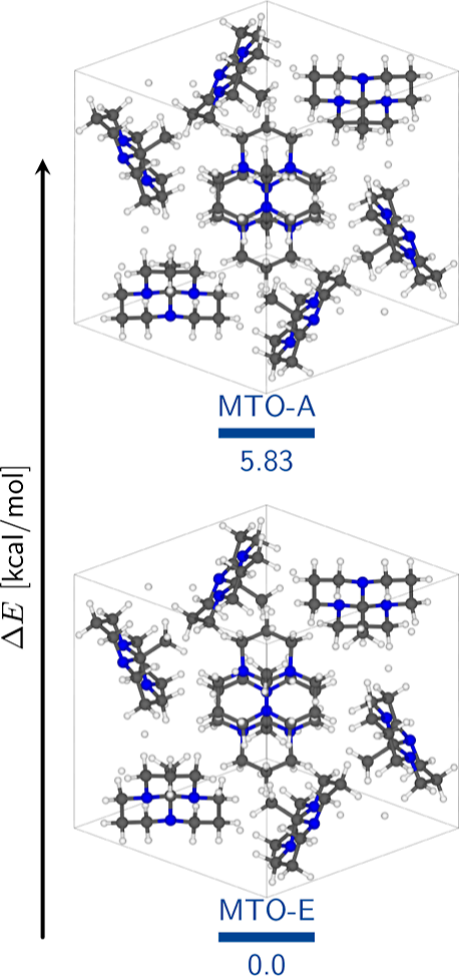
Energy difference (Δ*E*) between crystals with the all-*trans* orthoamide in its alternate and eclipsed conformations. Crystal systems hold the symmetry of the HTO crystal (water molecules were removed).

When the orthoamide molecule exists in the gas phase and solution, based on the same analysis as above, our thermodynamic computational study (refer to the Supporting Information) demonstrates that the eclipsed conformation of the methyl groups represents a transition state in forming the staggered structure. This conformation is not an energy minimum and lacks stability. As a result, it is highly improbable to observe both the alternated and eclipsed conformations in equilibrium. This explains why only the methyl group’s alternated conformation is found in the ATO crystal (Figure 3a,b). On the other hand, when the orthoamide molecule is surrounded by water molecules, hydrogen bond interactions (*r*_O···H_ = 2.55 Å and *∠*_C–H···O_ = 172.89°, to be discussed later) promote the eclipsing of the methyl group (Figure 3c). However, upon removal of the water molecules from the HTO crystal (MTO-A and MTO-E systems) and optimization of the positions of all hydrogen atoms and the methyl group’s carbon atom, we discovered that both conformations correspond to local minima, with a difference of approximately 6 kcal/mol between them (Figure 4). Consequently, both water molecules and the crystalline environment (characterized by interactions with neighboring molecules in a confined space compared to the liquid and gaseous states) appear to modify the molecular potential energy surface (PES) favoring the eclipsed conformation in the all-*trans* tricyclic orthoamide.

**Figure 5:**
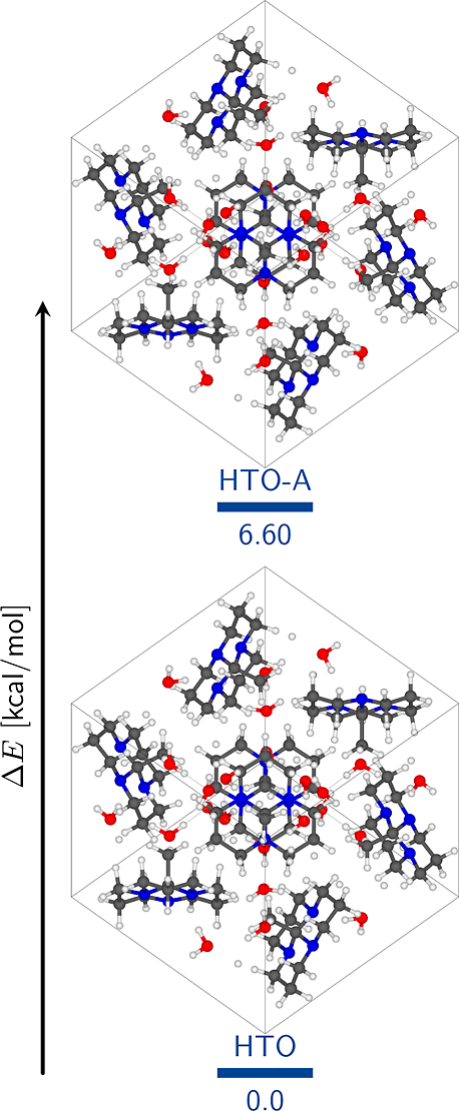
Energy difference (Δ*E*) between hydrated crystals with the all-*trans* orthoamide in its alternate and eclipsed conformations. Crystal systems hold the symmetry of the HTO crystal (water molecules were conserved).

On the other hand, we alternated the methyl group within the HTO system to examine its impact on the crystal system’s stability. This resulted in a new arrangement called the HTO-A system, where we optimized the positions of the hydrogen atoms. However, we did not change the coordinates of the water molecule oxygens as the LPs of nitrogen anchor them. The optimization process revealed that the methyl group remained closely alternated without being eclipsed , and we observed that the eclipsed conformation exhibited greater stability compared to its alternate counterpart when analyzing the energies of both the HTO crystal and the HTO-A system (Figure 5). This suggests that the eclipsed conformation is the preferred arrangement in a hydrated environment due to the interactions established by the methyl group. We will discuss this further later on.

### 3.2 Electron Density and Noncovalent Interaction Analysis

The molecular geometry analysis revealed that the conformation adopted by the tricyclic orthoamide in a specific environment (anhydrous and hydrated) is influenced by its interaction with water molecules and its contact with neighboring orthoamide molecules. We performed an electron density analysis of the studied systems using the QTAIM and NCI tools to gain a comprehensive understanding.

**Figure 6:**
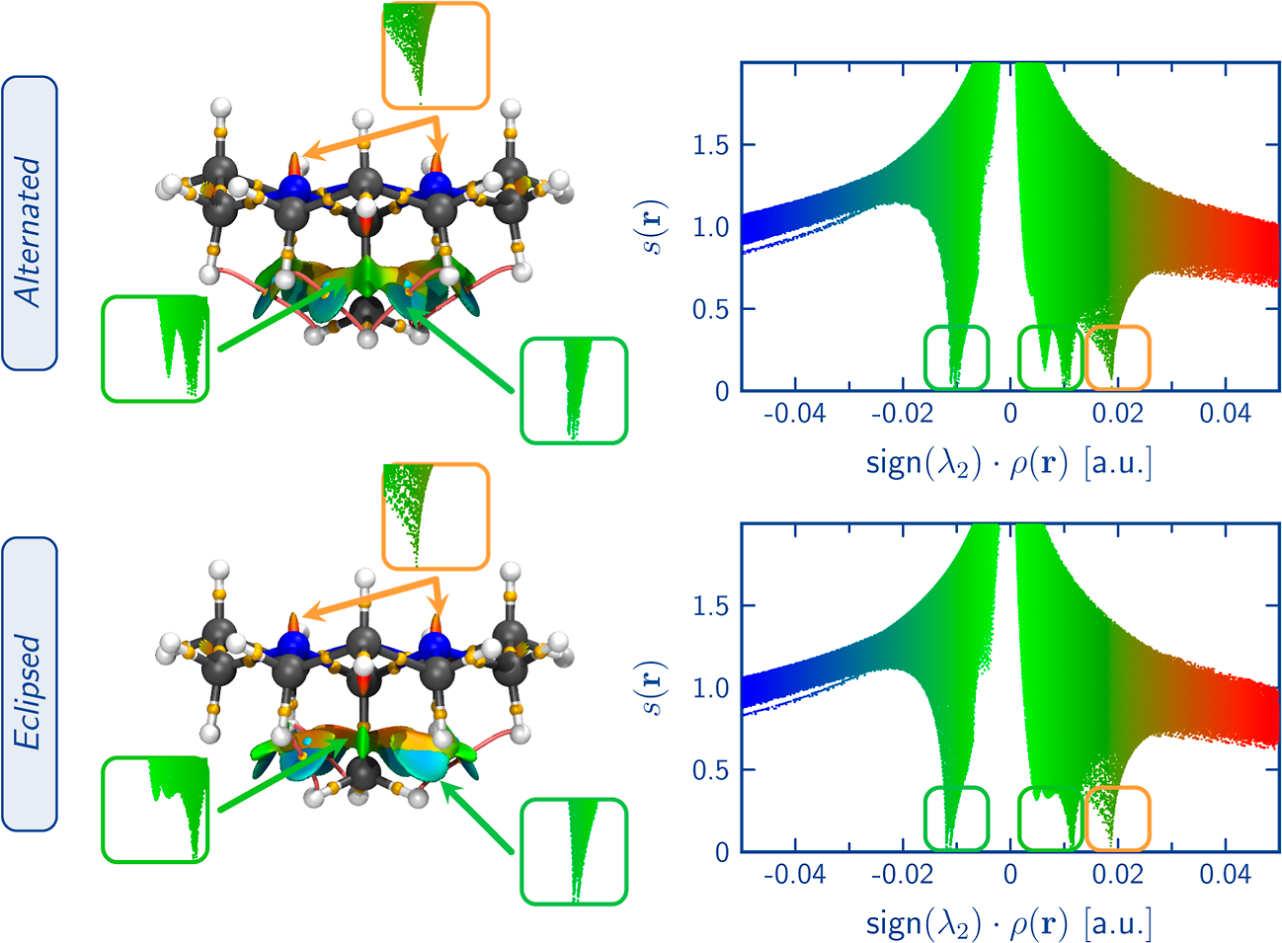
Alternated and eclipsed orthoamides’ BCP and RCP with their corresponding BP and NCI plots and surfaces. The NCI surfaces (isovalue = 0.05 au) are color-coded on a scale from −2.00 a.u. (blue) to 2.00 a.u. (red). BCP and RCP correspond to the gold and cyan spheres, respectively. The pink trajectories represent BP. Properties of CPs are reported in Table S15 and the Supporting Information.

Figure 6 showcases the BCPs and RCPs and the NCI surfaces of the isolated molecules of alternated and eclipsed orthoamides obtained from ATO and HTO crystals, respectively. A summary of the topological properties of BCPs associated with weak interactions is shown in Table 1 (more detailed information can be found in Table S15 in the Supporting Information). BCPs associated with the interaction between two hydrogen atoms (H–H bonding^48^) are present in both cases. The properties of these BCPs indicate that these interactions are crucial since ρ > 0.01 au, they are *closed-shell* (noncovalent), as indicated by a positive Laplacian value (average Laplacian of the interactions in the alternated and eclipsed conformers are 0.0441 and 0.0488 au, respectively). Moreover, analyzing the BD, (lower BD value corresponds to a stronger interaction^49^) it is noticeable that the eclipsed conformer exhibits very slightly stronger H–H bonds compared to the alternated conformer (BD average values for the eclipsed and alternated conformer are 0.1884 and 0.2048 au, respectively, see Table 1). Nevertheless, the latter has more such interactions, whose cooperative effect makes the alternate conformer more stable. According to the NCI surfaces, the H–H bonding interactions are characterized by strong attractive (the blue region), weak attractive (the green region), and nonattractive (the red-orange region) fractions. The alternated conformation demonstrates more attractive regions and fewer nonattractive regions than the eclipsed structure. This disparity contributes to the alternated conformation’s lower energy and local minimum nature in the isolated molecule’s PES. However, it is crucial to note that the attractive regions in the eclipsed orthoamide are not negligible. Therefore, under appropriate conditions, such as in a suitable environment such as the crystalline phase, the eclipsed conformation may exhibit stability and be a minimum in the PES.

**Table 1:** Topological Analysis of Electron Density in BCPs for H–H Interactions^a^

	**alternated** conformer					**eclipsed** conformer
	ρ(**r**)	∇^2^ρ(**r**)	*H*(**r**)/ρ(**r**)	ρ(**r**)	∇^2^ρ(**r**)	H(r)/ρ(r)
1	0.0105	0.0435	0.2072	0.0120	0.0488	0.1884
2	0.0104	0.0427	0.2076	0.0120	0.0488	0.1884
3	0.0111	0.0451	0.1955	0.0120	0.0488	0.1884
4	0.0112	0.0456	0.1953			
5	0.0102	0.0434	0.2183			
average	0.0107	0.0441	0.2048	0.0120	0.0488	0.1884

a Electron density, ρ(**r**), its Laplacian, ∇^2^ρ(**r**), and bond degree, BD = *H*(**r**)/ρ(**r**), are reported in atomic units.

**Figure 7:**
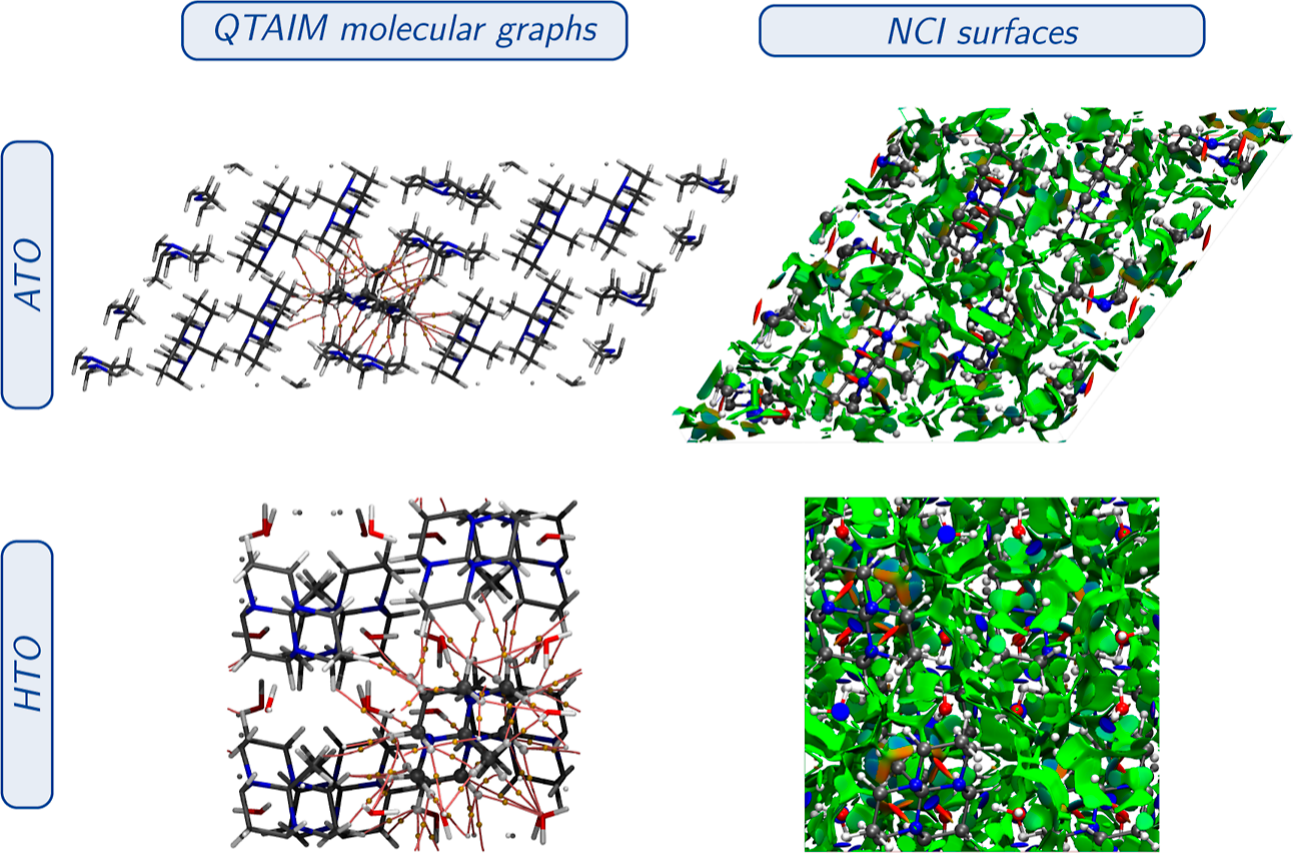
Noncovalent interactions between BCPs (with their corresponding BP) and NCI surfaces of the optimized ATO and HTO crystals. Gold spheres represent BCPs, whereas pink trajectories correspond to BPs. For the ATO QTAIM analysis, we used the 2 × 2 × 1 supercell representation since, in the asymmetric unit, there is not a complete all-*trans* tricyclic orthoamide. For both cases, the all-*trans* orthoamide is used as a reference. The NCI surfaces (isovalue = 0.05 a.u.) are color-coded on a scale from −2.00 a.u. (blue) to 2.00 a.u. (red).

Supporting the above-mentioned observations, Figure 7 displays the BCPs associated with noncovalent interactions and the corresponding NCI surfaces of the ATO and HTO crystals of tricyclic orthoamide. Both the ATO and HTO crystals exhibit various types of noncovalent interactions, covering hydrogen bonding, H–H bonding, and dispersion interactions. In the ATO crystal, the hydrogen bonds observed are of the C–H···N type, involving the nonbonding LP at orthoamide nitrogens and the neighboring orthoamide hydrogens. In the HTO crystal, the hydrogen bonding interactions consist of the O–H···N and C–H···O types. These interactions occur between the LPs of the orthoamide nitrogens and the water molecule hydrogens and between the LPs of the water molecule oxygen and the methyl group’s hydrogens. Furthermore, both systems display dispersion interactions, indicating the presence of weak (dispersion) intermolecular forces. Therefore, the stabilization of the eclipsed methyl group in HTO can be attributed to the abundance of stronger noncovalent interactions, particularly hydrogen bonds, present in the crystal structure.

**Figure 8:**
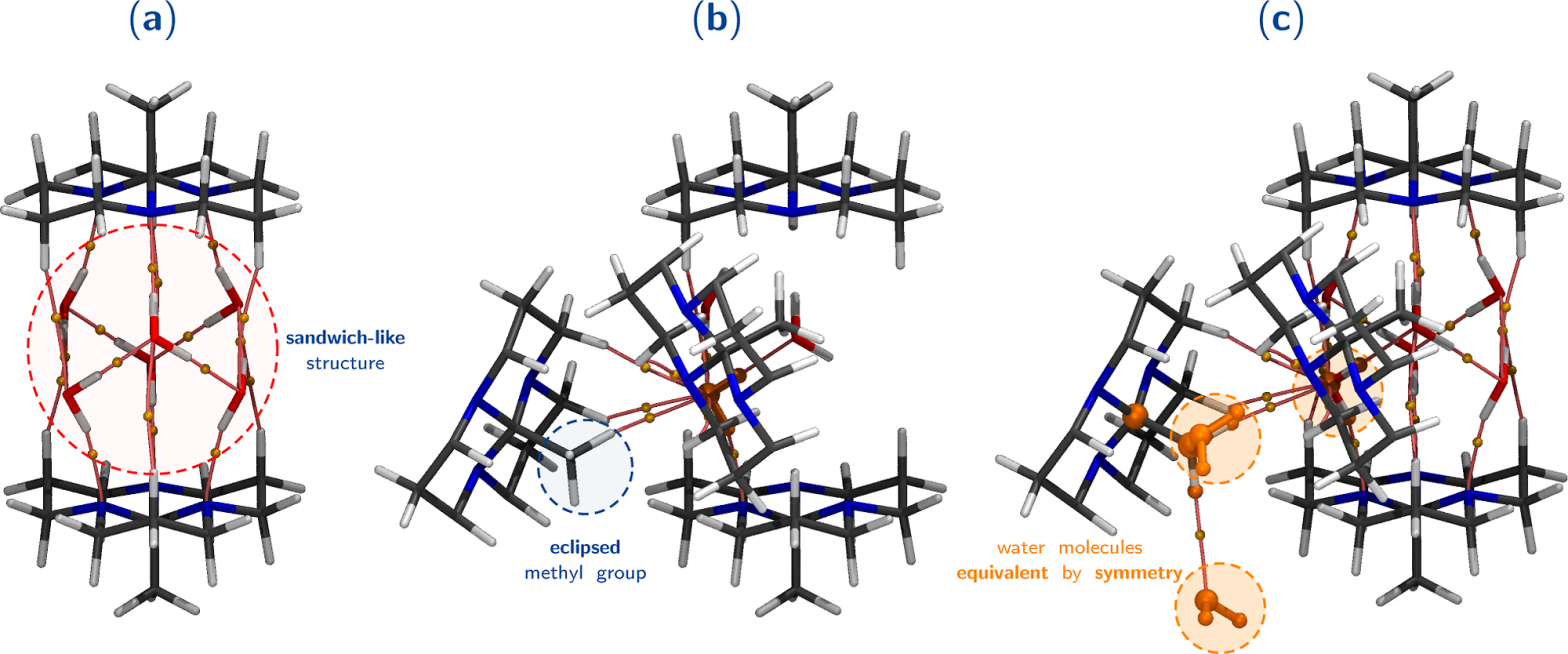
(a) Sandwich-like structure formed with a cluster of six interacting water molecules and two all-*trans* orthoamide molecules. (b) Each water molecule interacts with the LP of a nitrogen atom from the orthoamide tricycle and a hydrogen atom from the methyl group of another orthoamide molecule located outside the sandwich structure. (c) Symmetrical fraction is highlighted in orange. We extracted this structure from the optimized HTO crystal.

It is important to highlight that the HTO system exhibits a distinctive arrangement in which six water molecules form a network through hydrogen bonds between two parallel all-*trans* orthoamide molecules, forming a sandwich-like structure. This network is illustrated in Figure 8a. Each water molecule interacts with the nonbonding LP at the nitrogen atom from the orthoamide tricycle, two water molecules in the network confined by the two orthoamides, and hydrogen atoms of two other orthoamide molecules located outside the sandwich structure (Figure 8b). Due to the crystal’s symmetry, one water molecule (highlighted in orange in Figure 8c) occupies the same position in other water clusters (i.e., sandwich structures like the one shown in Figure 8a) that can be formed throughout the crystal (indicated by the orange fraction highlighted in Figure 8c). This specific arrangement between the water molecules and the orthoamide molecules, along with the higher interaction energy of the hydrogen bonds observed in the HTO crystal compared to ATO (to be discussed later), contributes to the restriction that only the all-*trans* structure is present for the system in which water molecules are present (HTO crystal). This observation aligns with the findings from the geometry and energy analyses.

**Figure 9:**
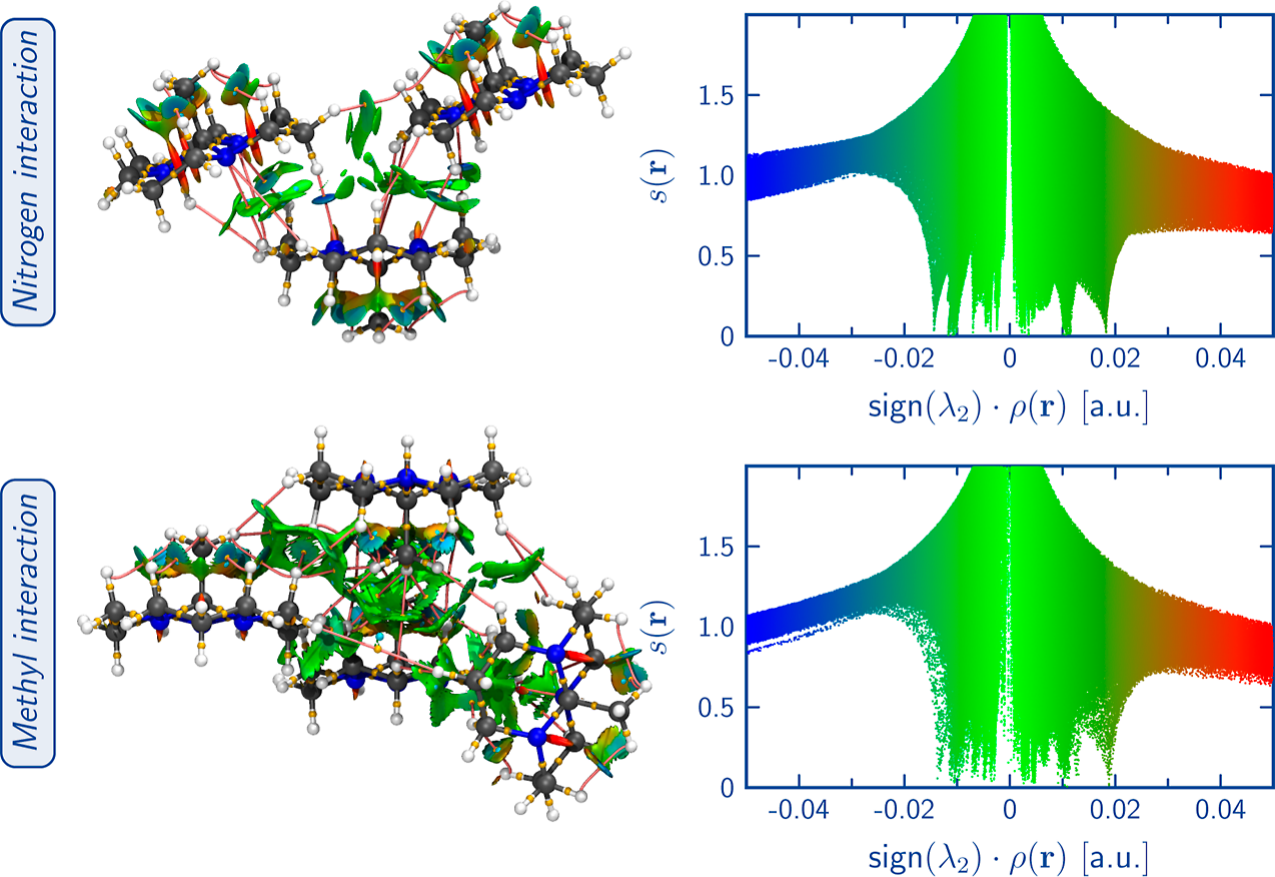
Interactions associated with the nitrogen’s LPs (top) and methyl group (bottom) of the all-*trans* alternated orthoamide in the ATO crystal environment. BCPs (gold spheres), RCPs (cyan spheres), and NCI surfaces are shown. The NCI surfaces (isovalue = 0.05 a.u.) are color-coded on a scale from −2.00 a.u. (blue) to 2.00 a.u. (red).

**Figure 10:**
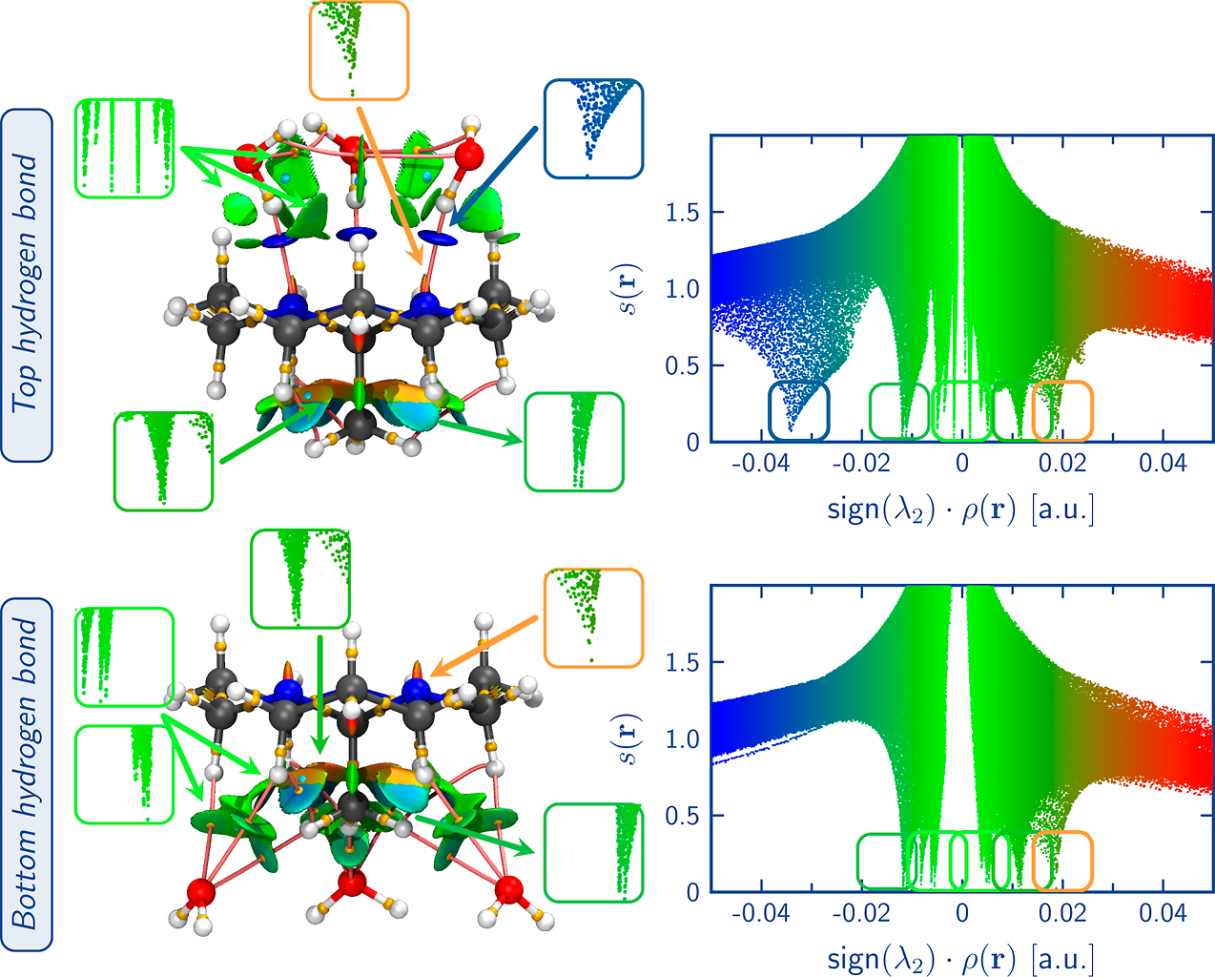
Interactions between nitrogens’ LPs and water molecules (top) and methyl group and water molecules (bottom) of the all-*trans* eclipsed orthoamide in the HTO crystal environment. BCPs (gold spheres), RCPs (cyan spheres), and NCI surfaces are shown. The NCI surfaces (isovalue = 0.05 a.u.) are color-coded on a scale from −2.00 a.u. (blue) to 2.00 a.u. (red).

Figures 9 and 10 depict the interactions involving the nitrogens’ LPs and the methyl group of the tricyclic orthoamide in the ATO and HTO crystals, respectively. The analysis was conducted using QTAIM and NCI methods, focusing on the electron density of the corresponding fractions. Regarding the interactions with nitrogens, both systems can form hydrogen bonds, as evidenced by the presence of the classic disk on the NCI surface^50^ and the corresponding BCP. Qualitatively, the NCI analysis reveals that the hydrogen bonds of the O–H···N type formed in the HTO crystal are stronger than the C–H···N type interactions observed in ATO. This observation is supported by the density values at the BCP and the BD. The average density values for the C–H···N and O–H···N interactions are 0.0077 and 0.0346 a.u., respectively. Notably, the interaction of O–H···N exhibits a higher average density value, denoting its greater strength. This observation aligns with the average BD values of 0.0218 a.u. for C–H···N and −0.0914 a.u. for O–H···N interactions, with the O–H···N interaction displaying the lowest BD value, reinforcing its stronger bonding characteristics (see Table 2 and for detailed information, Tables S16–S18, in Supporting Information). Similarly, interactions involving the methyl group of the orthoamides exhibit stronger interactions in HTO compared to ATO. In ATO, these interactions correspond to H–H bonds (C–H···H, with average values of density and BD at the BCP of 0.0094 and 0.1959 a.u., respectively), whereas in HTO, they are hydrogen bonds (C–H···O, with average values of 0.0083 and 0.1509 a.u. for density and BD at the BCP, respectively).

**Table 2:** Topological Analysis of Electron Density in BCPs for X–H···N Interactions, X = {C,O}, and for C–H···Y Interactions, Y = {H,O}. Electron Density, ρ(**r**), and Bond Degree, BD = *H*(**r**)/ρ(**r**), Are Reported in Atomic Units

	ATO’s system (Figure 9)				HTO’s system (Figure 10)			
	C–H···N		C–H···H^a^		O–H···N		C–H···O	
	ρ(**r**)	*H*(**r**)/ρ(**r**)	ρ(**r**)	*H*(**r**)/ρ(**r**)	ρ(**r**)	*H*(**r**)/ρ(**r**)	ρ(**r**)	*H*(**r**)/ρ(**r**)
1	0.0037	0.1575	0.0067	0.1874	0.0346	–0.0914	0.0083	0.1510
2	0.0024	0.2062	0.0104	0.2067	0.0346	–0.0914	0.0083	0.1509
3	0.0101	0.1204	0.0111	0.1936	0.0346	–0.0914	0.0083	0.1509
4	0.0145	0.0544						
average	0.0077	0.0218	0.0094	0.1959	0.0346	–0.0914	0.0083	0.1509

a Average values are shown for the ATO’s methyl group hydrogens since some of them have more than one associated BP. The properties related to all BPs can be found in Table S18 of the Supporting Information.

It is noteworthy that the intermolecular hydrogen bonds formed between water molecules and the LPs of nitrogens, as well as the intramolecular H–H bonds between the hydrogens of the methyl group and the tricycle orthoamide’s hydrogens in HTO, do not exert a significant effect (Table S16 in the Supporting Information). Conversely, anticooperative effects are observed, manifested by a decrease in the strength of H–H bonding interactions (Table S17 in the Supporting Information), resulting from the formation of hydrogen bonds between water molecules and the hydrogens of the methyl group. However, these effects are counterbalanced by the C–H···O hydrogen bonds formed between water molecules and the methyl group as well as between water molecules and the orthoamide tricycle. Overall, these interactions favor stabilization of the eclipsed conformation.

**Figure 11:**
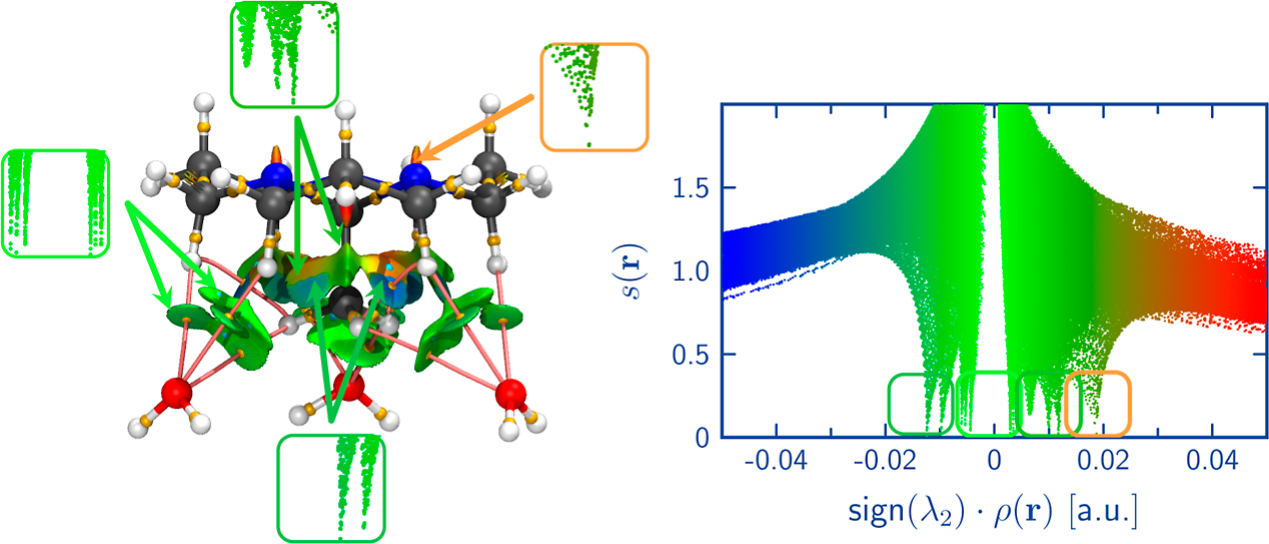
Interactions between the methyl group and water molecules of the all-*trans* alternated orthoamide in the HTO crystal environment. BCPs (gold spheres), RCPs (cyan spheres), and NCI surfaces are shown. The NCI surfaces (isovalue = 0.05 a.u.) are color-coded on a scale from −2.00 a.u. (blue) to 2.00 a.u. (red).

We can support the last observation by examining the segment of the HTO-A system that deals with the interaction between the orthoamide and the surrounding water molecules of the alternated methyl group. Figure 11 showcases the corresponding NCI and QTAIM analysis. Notably, we can observe an enhancement of the H–H bonding interactions, as shown by the prevalence of bluer regions on the associated surface, coupled with a decrease in BD values from 0.1981 a.u. in the HTO system to 0.1770 a.u. in the HTO-A system (Table S17 in the Supporting Information). However, in contrast, the O···H–C hydrogen bonds exhibit weakened strength compared to their counterparts within the eclipsed system. This is represented by the transition to a green color on the NCI surface compared to HTO, accompanied by an increase in BD values. Specifically, the interactions involving the methyl group’s hydrogens observe an increase in BD value from 0.1510 a.u. in the HTO system to 0.2031 a.u. in the HTO-A system. Similarly, the interactions that involve the axial hydrogens of the methylenes within the tricyclic orthoamide see a rise in BD values from 0.1695 a.u. in the HTO system to 0.1874 a.u. in the HTO-A system (Table S17 in Supporting Information). Therefore, in conjunction with the energy analysis, we can confirm that the system is destabilized due to the weakening of hydrogen bonds. This proves that the eclipsed conformation is preferred in a hydrated environment due to hydrogen interactions between water molecules and both the methyl group’s hydrogens (as concluded in previous works^11^) and the axial hydrogens of the methylenes of the fused rings, as demonstrated in this work.

It is worth noting that the interpretation of H–H BPs within the framework of QTAIM theory remains a topic of ongoing debate in the literature.^51−58^ Our analysis, based on X-ray experimental crystalline structures, reveals the presence of BCPs and NCI surfaces, suggesting the existence of H–H interactions. Furthermore, our results indicate their potential contribution to stabilizing the eclipsed conformer, which is in agreement with the existence of the eclipsed methyl group in the HTO system.

### 3.3 Interaction Energy Descomposition

**Figure 12:**
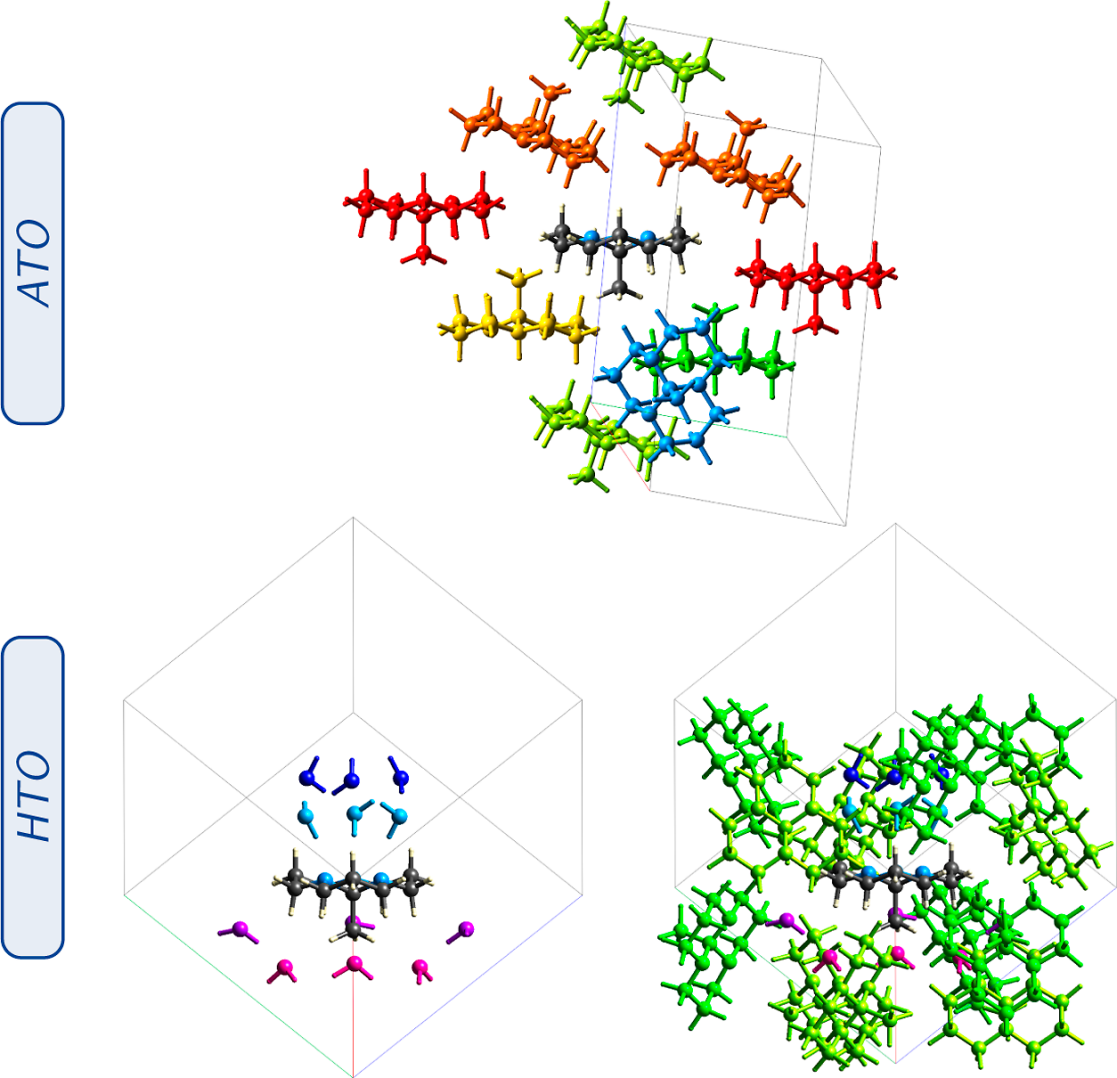
Principal interacting molecules in the ATO (top) and HTO (bottom) crystals. An all-*trans* orthoamide was used as a reference for both systems. Molecules are colored by their corresponding symmetry equivalences. Total interaction energies and their contributions, as well as all of the molecule clusters around the reference molecule, are reported in Tables S19 and S20 in the Supporting Information.

Lastly, to further reinforce the electron density analysis, we present a pairwise energetic analysis of the interactions between an all-*trans* orthoamide and its environment for both the anhydrous and the hydrated crystals. Figure 12 illustrates the most significant interacting neighbors of a reference orthoamide (either alternated or eclipsed) for both the ATO and HTO systems. See Tables S19 and S20 in the Supporting Information to view the contributions and magnitudes of the interaction energy. The molecules are color-coded based on their symmetry equivalence, providing additional insights into the intermolecular interactions within the crystal.

In the ATO system, most interacting molecules are in the all-*trans* configuration (top of Figure 12). The only *cis,cis,trans* molecule (highlighted in cyan) interacts weakly with the reference orthoamide (Table S19 in the Supporting Information), which is supported by the analysis of QTAIM (Table S18 in the Supporting Information) and NCI (Figure 9). The molecules highlighted in orange exhibit the strongest interactions with the reference orthoamide, primarily through hydrogen bonding and with substantial contributions of the electrostatic and dispersive terms to the total interaction energy (Table S19 in the Supporting Information). The yellow and green molecules interact significantly through H–H bonding and dispersive interactions, specifically with the methyl group of the reference orthoamide. The dispersive term plays a prominent role in the overall interaction energy in these interactions (Table S19 in the Supporting Information). The remaining red and green-yellow molecules surrounding the reference orthoamide interact through H–H bonds and dispersion interactions with the hydrogens of the tricycle, where the dispersive term is the primary contributor to stabilizing these interactions (Table S19 in the Supporting Information).

In the case of HTO (bottom of Figure 12), the eclipsed all-*trans* orthoamide exhibits the strongest interaction with the cyan water molecules through hydrogen bonding. The electrostatic contribution is the most significant component in the overall interaction energy (Table S20 in the Supporting Information). The green-yellow orthoamides surrounding the reference orthoamide establish interactions through H–H bonding and dispersion interactions. In this case, the dispersion term is crucial in the interaction energy (Table S20 and Supporting Information). The interaction between the water molecules (highlighted in pink) and the methyl group occurs via hydrogen bonding. However, these interactions are relatively weaker, with the dispersive contribution being the primary factor in the interaction energy (Table S20 in the Supporting Information); this is reflected in the green regions observed on the NCI surface (Figure 10). Despite being more distant, the reference orthoamide molecule can still interact with other water molecules (highlighted in purple and navy blue, bottom of Figure 12). However, these interactions are primarily dispersive (Table S20 in the Supporting Information). The observed interactions align with the QTAIM (Tables S16 and S17 in the Supporting Information) and NCI (Figure 10) analysis findings, confirming the types and natures of the interactions within the HTO system.

According to the previous analysis, the ATO crystal exhibits predominantly dispersive interactions, while the HTO crystal displays significant contributions from both electrostatic and dispersive terms. The electrostatic contributions associated with hydrogen bonding play a crucial role in stabilizing the eclipsed all-*trans* conformation. The presence of water molecules in HTO limits the inversion of the all-*trans* configuration by strongly interacting with their nitrogen LPs. Additionally, the water molecules enable the eclipsing of the methyl group by interacting with both the hydrogens of the methyl group and the hydrogens of the tricycle. These combined interactions contribute to stabilization of the eclipsed conformation in HTO.

## 4 Conclusions

This study investigates noncovalent interactions in two crystalline systems of tricyclic orthoamide, HTO and ATO. Through a comparative analysis of both systems, we have shown that these interactions have a significant impact on the structural configuration of orthoamides and can stabilize energetically unfavorable conformations. The analysis of electron density and interaction energies reveals that hydrogen bond interactions, specifically, O–H···N, O–H···O, and C–H···O, are the strongest driving forces in the crystal molecular arrangement. Both systems also show significant contributions of H–H bonding (C–H···C–H) and dispersion interactions. Comparing HTO and ATO systems, we found that O–H···N interactions are particularly important, as they induce the orthoamide to predominantly adopt an all-*trans* configuration. Together with the O–H···O interaction, they create a unique arrangement of water molecules in the HTO system, forming cavities where the orthoamide methylenes intercalate. This leads to an intriguing arrangement where the hydrogen atoms of the methyl group experience a directional attractive force from the water molecules via C–H···O interaction, eclipsing the C–H and C–N bonds. This research provides valuable insights into molecular recognition in organic crystals and the mechanics governing the conformational dynamics. We hope this work will lead to novel molecular architectures with tailored molecular arrangements, as these arrangements directly influence the physical and chemical properties of the molecular systems.

## Data Availability

The data underlying this study are available in the published article and its Supporting Information.

## Supporting Information Available

Crystallography data, QTAIM and NCI analysis for ATO and HTO systems, inversion and conformational processes in tricyclic orthoamide, and topological analysis of electron density (PDF)
