# Supplementary material for: Revealing the Role of Noncovalent Interactions on the Conformation of the Methyl Group in Tricyclic Orthoamide
Source: J Org Chem. 2023 Dec 15;89(1):257–68. doi: 10.1021/acs.joc.3c02016 (PMC10921461; doi:10.1021/acs.joc.3c02016)
Supplement: Supplementary file 1 — jo3c02016_si_001.pdf [file jo3c02016_si_001.pdf]

Revealing the Role of Non-covalent Interactions on the Conformation of Methyl Group in Tricyclic Orthoamide

Supporting Information

Jorge Gutiérrez-Flores[1], Eduardo H. Huerta[2] ·
Gabriel Cuevas[2], Jorge Garza[1], Rubicelia Vargas[1,*]

rvargas@izt.uam.mx

[1]Departamento de Química, División de Ciencias Básicas e Ingeniería, Universidad Autónoma Metropolitana Iztapalapa, San Rafael Atlixco 186, Col. Vicentina, C.P. 09340 Iztapalapa, CDMX, México

[2]Insituto de Química, Universidad Nacional Autónoma de México, Circuito Exterior, Ciudad Universitaria, Alcaldía Coyoacán C.P. 04510, CDMX

Table of contents

S1 Crystallography data **2**

S2 QTAIM and NCI analysis for ATO and HTO systems **8**

S3 Inversion and conformational processes in tricyclic orthoamide **12**

S4 Topological analysis of electron density **28**

S4 .1 Interaction energy calculation 32

S1 Crystallography data

This research utilized the crystallographic data reported in previous works and obtained from the Cambridge Structural Database (CCDC) [1]. The following tables summarize the crystallographic parameters of the systems investigated in this study. The fractional coordinates presented in the tables correspond to those resulting from the optimization calculations.

Table S1: Crystallographic data for optimized hydrated (HTO) and anhydrous (ATO) tricyclic orthoamide.

		HTO				ATO		
CCDC no			1254424				1254426	
Empirical Formula			$C_{11}H_{21}N_3$,3(H_2O)				$C_{11}H_{21}N_3$	
Crystal system			cubic				monoclinic	
Space group			$Pa3$				$P21/c$	
a [Å]			13.719(3)				17.502	
b [Å]			13.719(3)				8.537	
c [Å]			13.719(3)				17.674	
α [deg]			90				90	
β [deg]			90				125.89	
γ [deg]			90				90	
Cell volume [Å^3]			2582.07				2139.39	
Frac. coord.		x	y	z		x	y	z
	O1	0.3957	0.4025	-0.3958	C1	0.0618	0.2596	-0.0968
	H1	0.3754	0.3804	-0.4619	N1	0.0722	0.3987	-0.1396
	H2	0.3915	0.4749	-0.3966	C2	0.1698	0.4423	-0.0987
	C1	0.3291	0.3291	0.3291	C3	0.2220	0.3056	-0.1041
	N1	0.3119	0.3124	0.4347	C4	0.2109	0.1605	-0.0614
	C2	0.2116	0.3365	0.4647	N2	0.1112	0.1295	-0.1064
	C3	0.1904	0.4427	0.4417	C5	0.0976	-0.0223	-0.0775
	C4	0.3349	0.2116	0.4633	C6	-0.0068	-0.0613	-0.1340
	C5	0.2634	0.2634	0.2634	C7	-0.0608	0.0730	-0.1296
	H3	0.2068	0.3226	-0.4566	N3	-0.0397	0.2202	-0.1558

Continue on the next page

Table S1: (Continued.)

H4	0.1579	0.2881	0.4293	C8	-0.0987	0.3454	-0.1593
H5	0.1137	0.4596	0.4581	C9	-0.0851	0.4940	-0.1973
H6	0.2362	0.4900	0.4875	C10	0.0188	-0.4654	-0.1444
H7	0.3191	0.2048	-0.4585	C11	0.1008	0.2880	0.0069
H8	0.2872	0.1589	0.4256	H1	0.1680	-0.4570	-0.1385
H9	0.2111	0.2210	0.3066	H2	0.2073	0.4820	-0.0250
N1A	0.4347	0.3119	0.3124	H3	0.2971	0.3343	-0.0678
N1B	0.3124	0.4347	0.3119	H4	0.1916	0.2815	-0.1778
C4B	0.2116	0.4633	0.3349	H5	0.2512	0.1739	0.0155
C3A	0.4417	0.1904	0.4427	H6	0.2408	0.0578	-0.0731
H9A	0.3066	0.2111	0.2210	H7	0.1348	-0.1106	-0.0900
H9B	0.2210	0.3066	0.2111	H8	0.1300	-0.0258	-0.0015
C2A	0.4647	0.2116	0.3365	H9	-0.0171	-0.1690	-0.1069
C4A	0.4633	0.3349	0.2116	H10	-0.0327	-0.0829	-0.2067
C2B	0.3365	0.4647	0.2116	H11	-0.0456	0.0800	-0.0592
H7B	0.2048	-0.4585	0.3191	H12	-0.1373	0.0534	-0.1789
H8B	0.1589	0.4256	0.2872	H13	-0.1725	0.3060	-0.2044
H5A	0.4581	0.1137	0.4596	H14	-0.0832	0.3676	-0.0895
H6A	0.4875	0.2362	0.4900	H15	-0.1137	0.4775	-0.2710
H3A	-0.4566	0.2068	0.3226	H16	-0.1235	-0.4079	-0.1933
H4A	0.4293	0.1579	0.2881	H17	0.0469	-0.4192	-0.0738
C3B	0.4427	0.4417	0.1904	H18	0.0270	-0.3725	-0.1823
H7A	-0.4585	0.3191	0.2048	H19	0.0645	0.3853	0.0139
H8A	0.4256	0.2872	0.1589	H20	0.1760	0.3173	0.0487
H3B	0.3226	-0.4566	0.2068	H21	0.0921	0.1835	0.0372
H4B	0.2881	0.4293	0.1579	C12	-0.4275	0.3024	-0.3129
H5B	0.4596	0.4581	0.1137	N4	-0.4347	0.3904	-0.3885
H6B	0.4900	0.4875	0.2362	C13	-0.4461	0.2863	-0.4609
				C14	-0.3679	0.1633	-0.4212
				C15	-0.3603	0.0756	-0.3423
				N5	-0.3494	0.1842	-0.2725
				C16	-0.3368	0.0949	-0.1950

Continue on the next page

Table S1: (Continued.)

C17	-0.3194	0.2036	-0.1181
C18	-0.3963	0.3289	-0.1597
N6	-0.4088	0.4117	-0.2393
C19	-0.3300	-0.4805	-0.2061
C20	-0.3480	-0.3853	-0.2874
C21	-0.3582	-0.4979	-0.3588
C22	0.4751	0.2289	-0.3543
H22	0.4851	0.2275	-0.4979
H23	-0.4493	0.3614	0.4867
H24	-0.3001	0.2197	-0.3947
H25	-0.3829	0.0812	-0.4765
H26	-0.2988	-0.0033	-0.3069
H27	-0.4232	-0.0006	-0.3710
H28	-0.3989	0.0195	-0.2193
H29	-0.2762	0.0160	-0.1679
H30	-0.3180	0.1362	-0.0643
H31	-0.2498	0.2590	-0.0839
H32	-0.3797	0.4174	-0.1068
H33	-0.4634	0.2753	-0.1815
H34	-0.3249	-0.4047	-0.1530
H35	-0.2620	0.4571	-0.1724
H36	-0.2894	-0.3037	-0.2640
H37	-0.4132	-0.3165	-0.3185
H38	-0.2898	0.4429	-0.3284
H39	-0.3766	-0.4356	-0.4218
H40	0.4528	0.1521	-0.4135
H41	0.4238	0.3242	-0.3782
H42	0.4741	0.1568	-0.3036

Table S2: Crystallographic data for modified tricyclic orthoamide crystal.

		MTO-A				MTO-E	
Empirical Formula			$C_{11}H_{21}N_3$				$C_{11}H_{21}N_3$	
Crystal system			cubic				cubic	
Space group			$Pa3$				$Pa3$	
a [Å]			13.719				13.719	
b [Å]			13.719				13.719	
c [Å]			13.719				13.719	
α [deg]			90				90	
β [deg]			90				90	
γ [deg]			90				90	
Cell volume [Å^3]			2582.07				2582.07	
Frac. coord.		x	y	z		x	y	z
	C1	0.3291	0.3291	0.3291	C1	0.3291	0.3291	0.3291
	N1	0.3119	0.3124	0.4347	N1	0.3119	0.3124	0.4347
	C2	0.2116	0.3365	0.4647	C2	0.2116	0.3365	0.4647
	C3	0.1904	0.4427	0.4417	C3	0.1904	0.4427	0.4417
	C4	0.3349	0.2116	0.4633	C4	0.3349	0.2116	0.4633
	C5	0.2635	0.2635	0.2635	C5	0.2628	0.2628	0.2628
	H3	0.2068	0.3232	-0.4563	H3	0.2065	0.3227	-0.4564
	H4	0.1566	0.2885	0.4301	H4	0.1566	0.2888	0.4294
	H5	0.1142	0.4607	0.4592	H5	0.1142	0.4607	0.4591
	H6	0.2380	0.4890	0.4865	H6	0.2380	0.4891	0.4865
	H7	0.3202	0.2049	-0.4583	H7	0.3196	0.2046	-0.4584
	H8	0.2874	0.1576	0.4266	H8	0.2878	0.1576	0.4258
	H9	0.1864	0.2716	0.2822	H9	0.2106	0.2204	0.3061
	N1A	0.4347	0.3119	0.3124	N1A	0.4347	0.3119	0.3124
	N1B	0.3124	0.4347	0.3119	N1B	0.3124	0.4347	0.3119
	C4B	0.2116	0.4633	0.3349	C4B	0.2116	0.4633	0.3349
	C3A	0.4417	0.1904	0.4427	C3A	0.4417	0.1904	0.4427
	H9A	0.2822	0.1864	0.2716	H9A	0.3061	0.2106	0.2204
	H9B	0.2716	0.2822	0.1864	H9B	0.2204	0.3061	0.2106
	C2A	0.4647	0.2116	0.3365	C2A	0.4647	0.2116	0.3365

Continue on the next page

Table S2: (Continued.)

C4A	0.4633	0.3349	0.2116	C4A	0.4633	0.3349	0.2116
C2B	0.3365	0.4647	0.2116	C2B	0.3365	0.4647	0.2116
H7B	0.2049	-0.4583	0.3202	H7B	0.2046	-0.4584	0.3196
H8B	0.1576	0.4266	0.2874	H8B	0.1576	0.4258	0.2878
H5A	0.4592	0.1142	0.4607	H5A	0.4591	0.1142	0.4607
H6A	0.4865	0.2380	0.4890	H6A	0.4865	0.2380	0.4891
H3A	-0.4563	0.2068	0.3232	H3A	-0.4564	0.2065	0.3227
H4A	0.4301	0.1566	0.2885	H4A	0.4294	0.1566	0.2888
C3B	0.4427	0.4417	0.1904	C3B	0.4427	0.4417	0.1904
H7A	-0.4583	0.3202	0.2049	H7A	-0.4584	0.3196	0.2046
H8A	0.4266	0.2874	0.1576	H8A	0.4258	0.2878	0.1576
H3B	0.3232	-0.4563	0.2068	H3B	0.3227	-0.4564	0.2065
H4B	0.2885	0.4301	0.1566	H4B	0.2888	0.4294	0.1566
H5B	0.4607	0.4592	0.1142	H5B	0.4607	0.4591	0.1142
H6B	0.4890	0.4865	0.2380	H6B	0.4891	0.4865	0.2380

Table S3: Crystallographic data for hydrated modified tricyclic orthoamide crystal.

			**HTO-A**	
Empirical Formula			$C_{11}H_{21}N_3$,3(H_2O)	
Crystal system			cubic	
Space group			$Pa3$	
a [Å]			13.719	
b [Å]			13.719	
c [Å]			13.719	
α [deg]			90	
β [deg]			90	
γ [deg]			90	
Cell volume [Å^3]			2582.07	
Frac. coord.		x	y	z
	O1	0.3957	0.4025	-0.3958
	H1	0.3755	0.3804	-0.4619

Continue on the next page

Table S3: (Continued.)

H2	0.3916	0.4749	-0.3967
C1	0.3291	0.3291	0.3291
N1	0.3119	0.3124	0.4347
C2	0.2116	0.3365	0.4647
C3	0.1904	0.4427	0.4417
C4	0.3349	0.2116	0.4633
C5	0.2632	0.2632	0.2632
H3	0.2074	0.3232	-0.4564
H4	0.1578	0.2876	0.4303
H5	0.1138	0.4596	0.4582
H6	0.2363	0.4899	0.4875
H7	0.3196	0.2050	-0.4584
H8	0.2868	0.1591	0.4259
H9	0.1874	0.2644	0.2877
N1A	0.4347	0.3119	0.3124
N1B	0.3124	0.4347	0.3119
C4B	0.2116	0.4633	0.3349
C3A	0.4417	0.1904	0.4427
H9A	0.2877	0.1874	0.2644
H9B	0.2644	0.2877	0.1874
C2A	0.4647	0.2116	0.3365
C4A	0.4633	0.3349	0.2116
C2B	0.3365	0.4647	0.2116
H7B	0.2050	-0.4584	0.3196
H8B	0.1591	0.4259	0.2868
H5A	0.4582	0.1138	0.4596
H6A	0.4875	0.2363	0.4899
H3A	-0.4564	0.2074	0.3232
H4A	0.4303	0.1578	0.2876
C3B	0.4427	0.4417	0.1904
H7A	-0.4584	0.3196	0.2050
H8A	0.4259	0.2868	0.1591

Continue on the next page

Table S3: (Continued.)

H3B	0.3232	-0.4564	0.2074
H4B	0.2876	0.4303	0.1578
H5B	0.4596	0.4582	0.1138
H6B	0.4899	0.4875	0.2363

S2 QTAIM and NCI analysis for ATO and HTO systems

This section presents a more detailed view of Figures 2 and 7, originally included in the article. The purpose of including these figures is to highlight specific structural details that may require more clarity or emphasis in the versions presented in the main body of the work.

(**a**) Hydrated orthoamide crystal (HTO)

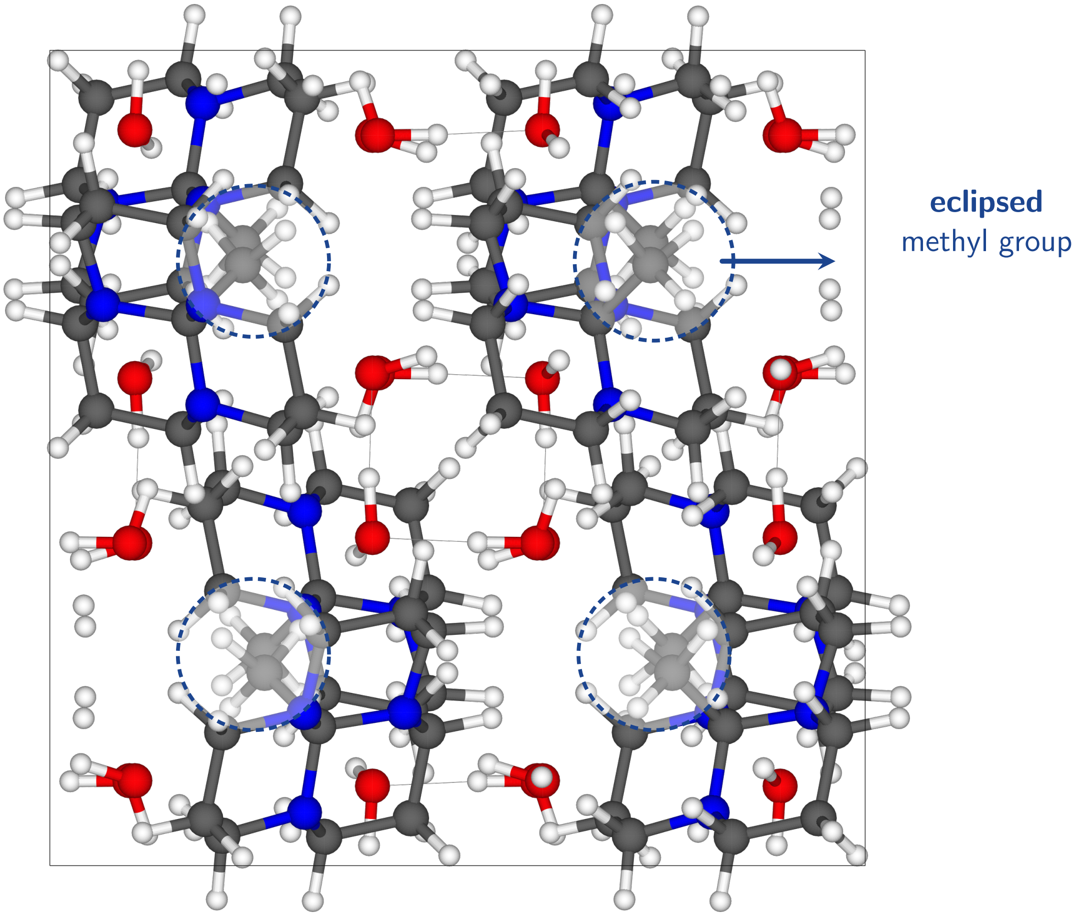

(**b**) Anhydrous orthoamide crystal (ATO)

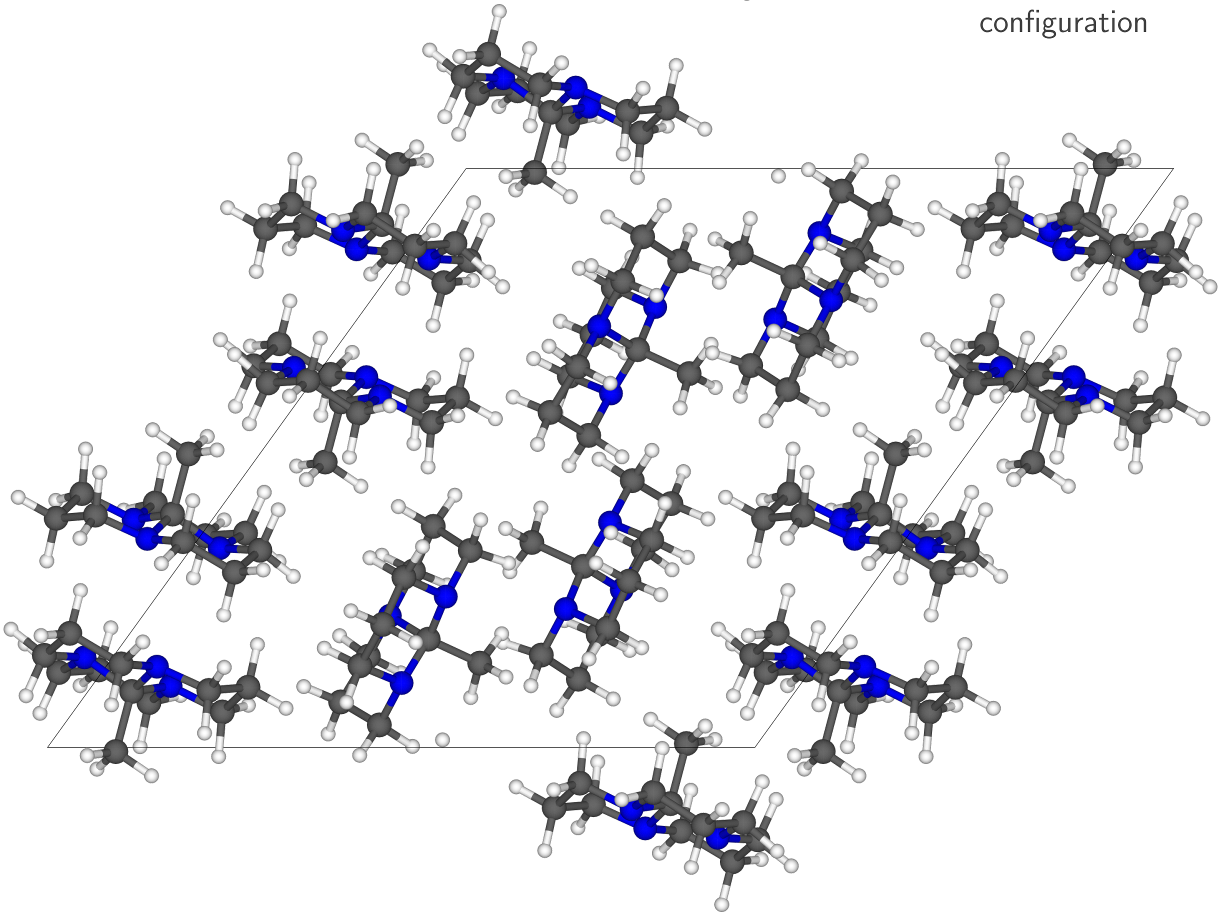

QTAIM and NCI for **ATO** system

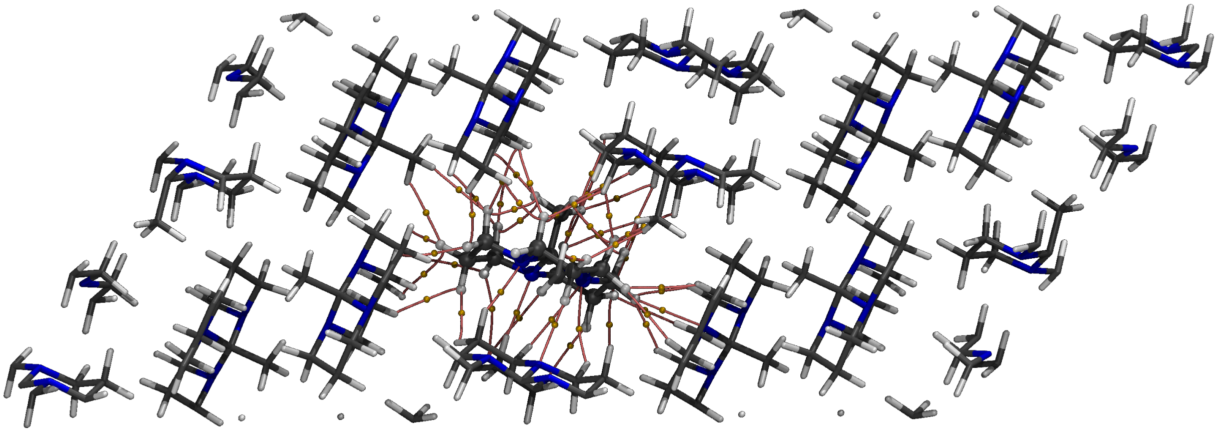

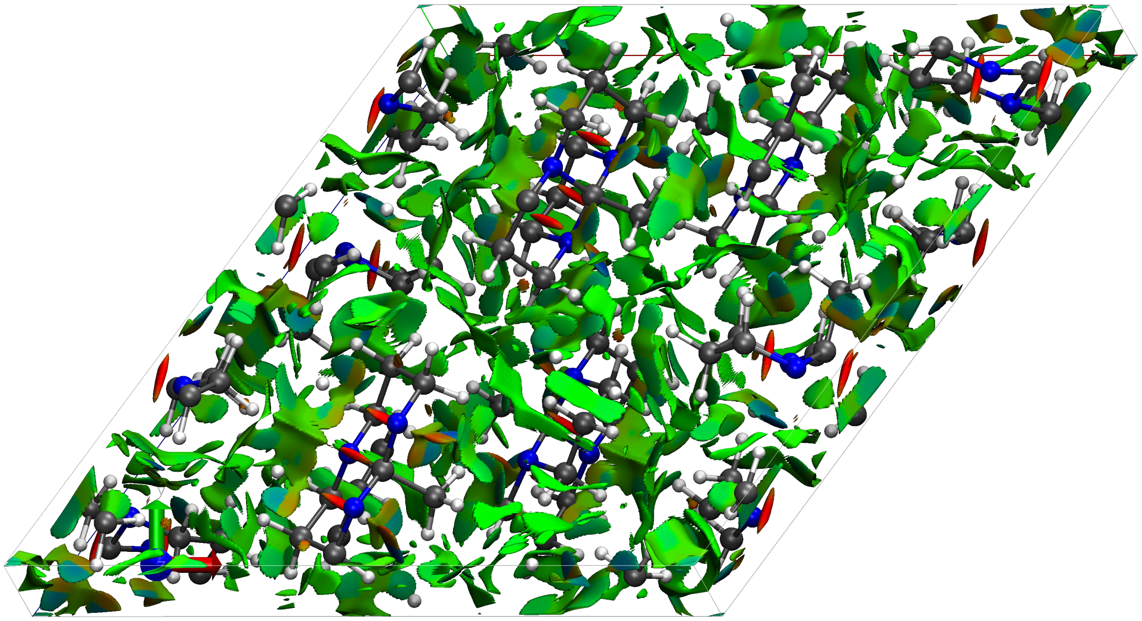

QTAIM and NCI for **HTO** system

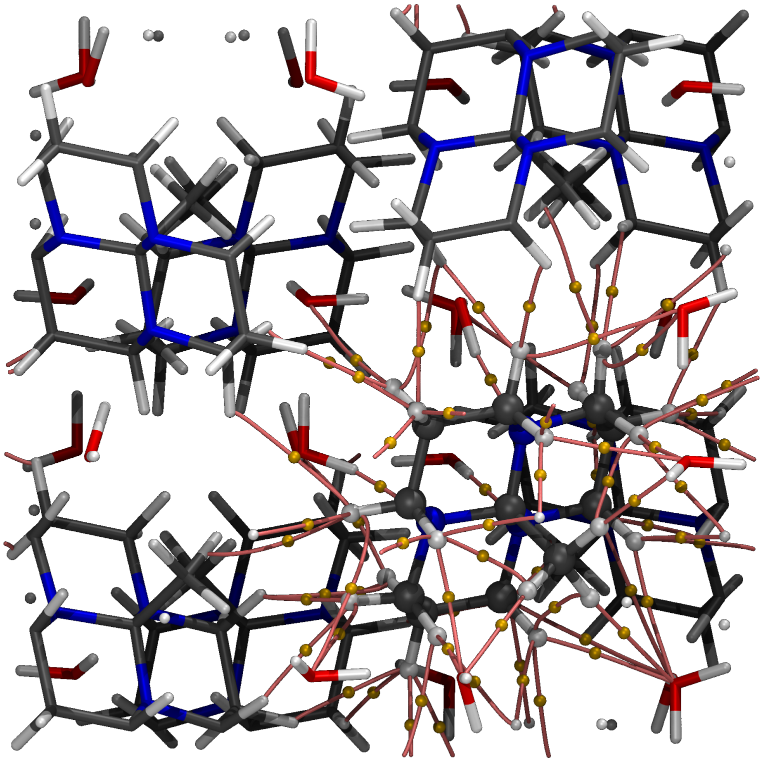

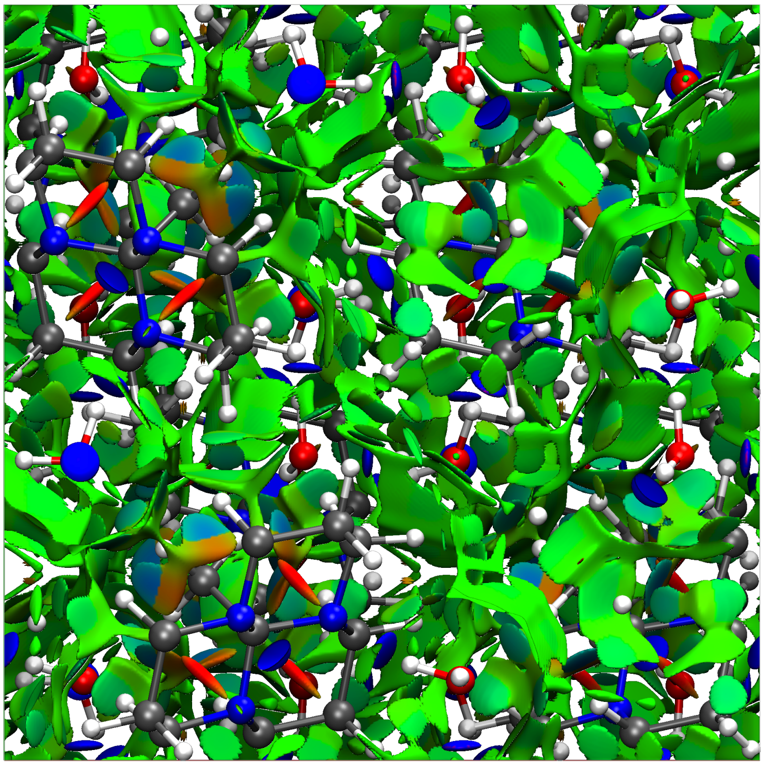

S3 Computational analysis of inversion and conformational processes in tricyclic orthoamide structure

Two essential processes within the architecture of tricyclic orthoamide deserve particular investigation. The first process, referred to as conformational inversion, involves the inversion of one of the three fused rings, resulting in the transition from the all-*trans* configuration to the *cis,cis,trans* configuration. The second process entails the conformational rotation of the methyl group attached to the central carbon of the tricyclic orthoamide. In order to analyze both processes, it is necessary to explore the potential energy surface (PES) of tricyclic orthoamide. With this in mind, this section is divided into three subsections. The first subsection introduces the theoretical approach employed to describe the molecular PES. The subsequent two subsections present the observations derived from the calculations for the inversion and conformational processes, respectively.

General methodology. The analysis was conducted employing the Gaussian16 program [2]. For describing the molecular potential energy surface (PES), we employed the B3LYP [3, 4, 5, 6] approximation within the Density Functional Theory (DFT) framework in conjucation with the 6–31G(d,p) basis set [7, 8]. Additionally, we considered the Grimme dispersion correction with Becke-Johnson damping [9] to account for the contributions from dispersion interactions not natively incorporated in the DFT framework. The solvent effect was simulate by employing the self-consistent reaction field (SCRF) theory integrating the solvation model based on density (SMD) proposed by Truhlar et al. [10]. In exploring the gas phase PES, we optimized the internal coordinates of the orthoamide for a set of torsion angles. Subsequently, the discovered stationary states were optimized without restrictions and confirmed their nature by calculating their frequencies. To incorporate the solvent effect, we performed single-point calculations using the optimized gas-phase geometry.

Inversion process. According to the crystallographic analysis reported by Dunitz et al. [1], the

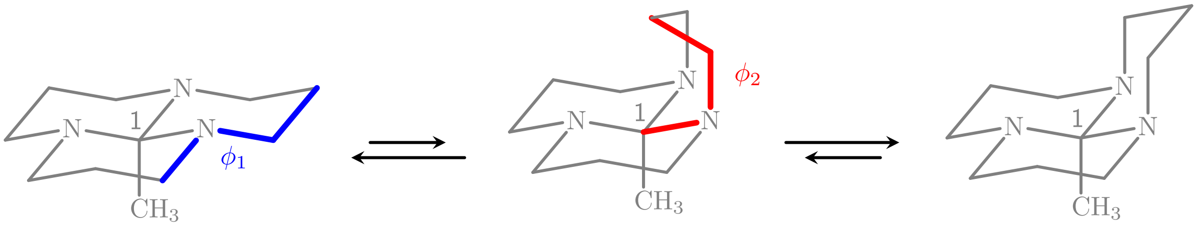

Figure S1: Steps performed to investigate the inversion process in tricyclic orthoamide from its all-trans to cis,cis,trans configuration.

tricyclic orthoamide exhibits two distinct stable configurations: all-*trans* and *cis,cis,trans*. The ^{13}C-NMR experiments conducted by Weisman et al. [11] at low temperatures suggest that the all-*trans* configuration is the most stable. To better understand the connection between these configurations, we investigated the inversion mechanism from the all-*trans* configuration to the *cis,cis,trans* configuration. This investigation was conducted in two stages (Figure S1). In the first stage, we optimized the internal coordinates of the orthoamide across a range of torsion angles ϕ_1(C–N–C–C). In the second stage, starting from the intermediate I-2, we optimized the internal coordinates for an other set of torsion angles ϕ_2(C1–N–C–C).

The inversion process from the all-*trans* to the *cis,cis,trans* configuration involves two intermediates (I-1 and I-2) and three transition states (TS1, TS2, and TS3) (Figure S2). In the initial step, which is endergonic, the ring undergoes a conformational change from a chair conformation to a twist-boat conformation (I-1 intermediate). Subsequently, in the second stage, the ring, which has acquired a twist-boat conformation, experiences an endothermic step to a boat conformation, resulting in I-2 intermediate. In the third and final stage, the ring, which is in the boat conformation, reverts back to its chair conformation through an exergonic step. It is important to note that the first step of the process is the limiting step.

The thermodynamic values (Table S4), including Gibbs free energy ($\Delta_{\text{inv}}G^{\circ}$) and equilibrium constant

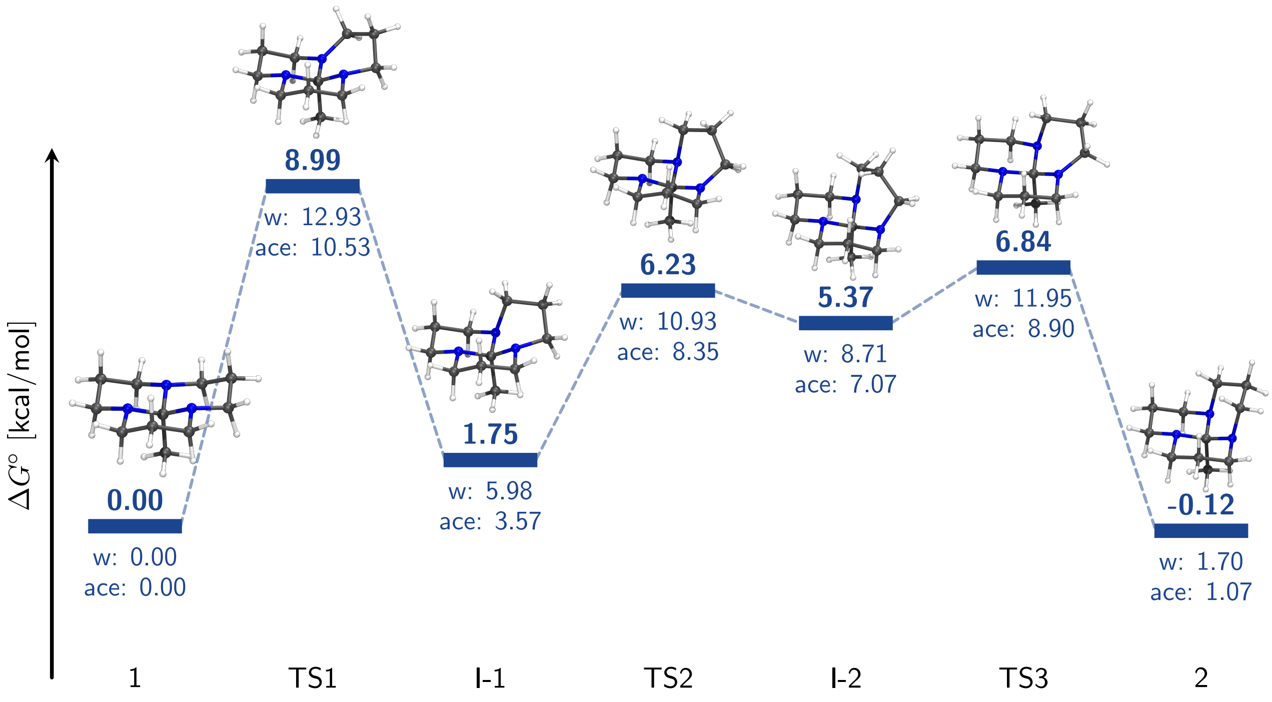

Figure S2: Energy profile illustrating the inversion process of tricyclic orthoamide. The diagram depicts relative energies in the gas phase (bold values on the bars) and in solution, considering water (w) and acetone (ace) as solvents.

(K), calculated in the gas phase, suggest a slight displacement of the configurational equilibrium towards the *cis,cis,trans* configuration. However, once the solvent effect is taken into account, the equilibrium direction is reversed, resulting in a preference for the all-*trans* configuration. It is essential to highlight that the thermodynamic and kinetic values indicate a rather fast equilibrium between both configuration and a high probability of the coexistence of them in the gas and solution phases.

Table S4: Thermodynamic Parameters of the Inversion Process. $\Delta_{\text{inv}}G^\circ$ values are reported in kcal/mol, and the equilibrium constant K are calculated with the equation $\Delta_{\text{inv}}G^\circ = -RT\ \ln K$.

	Gas		Water		Acetone	
	$\Delta_{\text{inv}}G^\circ$	K	$\Delta_{\text{inv}}G^\circ$	K	$\Delta_{\text{inv}}G^\circ$	K
**1** ⇄ **2**	-0.120	1.2245	1.70	0.0567	1.07	0.1643

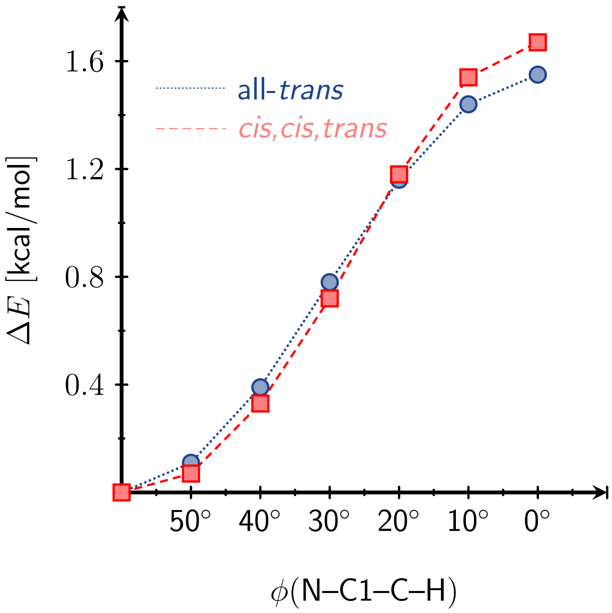

Figure S3: Energy profile of the rotation process of the methyl attached to the central carbon in the orthoamide in its both configuration: all-*trans* and *cis,cis,trans*.

Conformational process. The rotation of the methyl group attached to the central carbon of the tricyclic orthoamide is not free; a *rotational energy barrier* exists. To determine the magnitude of this barrier using the theoretical approach employed in this study, we optimized the internal coordinates of the tricyclic orthoamide for a specific set of torsion angles ϕ(N–C1–C–H) (Figura S3). The results reveal that both configurations exhibit a rotational barrier when transitioning from a *staggered conformation*, characterized by a torsion angle of 60 degrees, to an *eclipsed conformation*, characterized by a torsion angle of 0 degrees. Notably, the orthoamide in its *cis,cis,trans* configuration displays a higher rotational barrier.

The staggered and the eclipsed conformations represent stationary states on the PES; however, they differ in terms of their stability. The optimization performed in the gas phase and frequency calculations confirms that the staggered conformation is a minimum energy state and exhibits stability. In contrast, the eclipsed conformation corresponds to a maximum energy state, indicating instability. The evaluation of the rotation barrier in terms of the Gibbs free energy, both in the gas phase and in solution (Table

Table S5: Rotation free energy ($\Delta_{\text{rot}}G^{\ddagger}$) calculated by finding the difference between the Gibbs free energies of the eclipsed (transition state) and alternated (minimum energy state) conformers, in both orthoamide configuration: all-*trans* (**1**) and *cis,cis,trans* (**2**).

	$\Delta_{\text{rot}}G^{\ddagger}$ [kcal/mol]		
	Gas phase	Water	Acetone
**1**	1.76	1.56	1.84
**2**	1.97	2.01	2.09

S5), reveals that the energy cost associated with the rotation process needs to be more significant to anchor the methyl group to a single configuration.

The tables below display energy and structural data for stationary states resulting from inversion and conformational investigation.

**1**-staggered				
G (gas phase) [Hartrees]		-595.859381		
G (SMD-w) [Hartrees]		-595.873274		
G (SMD-ace) [Hartrees]		-595.876177		
Num. Im. Freq.		0		
Opt Coord		x	y	z
	C	-0.0290	-0.0033	0.0157
	C	-0.0112	-0.0539	1.5376
	C	1.4270	-0.0031	2.0351
	C	2.2252	-1.0024	-0.0746
	H	-0.5880	0.7796	1.9534
	H	0.2625	1.0073	-0.3314
	H	-1.0447	-0.1749	-0.3566
	H	1.8488	1.0073	1.8679
	H	1.4594	-0.1744	3.1165

Continue on the next page

H	-0.4669	-0.9928	1.8663
C	3.5268	-1.2022	2.0119
H	3.3808	-1.2721	3.0953
H	4.1798	-0.3236	1.8424
C	4.2061	-2.4614	1.4904
H	5.2238	-2.5382	1.8887
H	3.6348	-3.3345	1.8198
C	4.2335	-2.4354	-0.0318
H	4.5962	-3.3933	-0.4200
H	4.9494	-1.6666	-0.3831
C	0.6571	-1.2019	-1.9692
H	-0.4168	-1.2718	-2.1732
H	1.0244	-0.3235	-2.5353
C	1.3664	-2.4612	-2.4486
H	1.3102	-2.5383	-3.5401
H	0.8734	-3.3343	-2.0106
C	2.8193	-2.4353	-1.9935
H	3.3789	-1.6665	-2.5616
H	3.3023	-3.3932	-2.2150
N	0.8176	-1.0577	-0.5278
N	2.2101	-1.0579	1.4039
N	2.8861	-2.2377	-0.5511
C	2.9469	0.2728	-0.5948
H	2.4557	1.1820	-0.2434
H	3.9841	0.3115	-0.2567
H	2.9582	0.3094	-1.6856

TS1

G (gas phase) [Hartrees]	-595.845056
G (SMD-w) [Hartrees]	-595.852665
G (SMD-ace) [Hartrees]	-595.859393
Num. Im. Freq.	1

Continue on the next page

Opt Coord		x	y	z
	C	0.0297	-0.0150	0.0157
	C	0.1075	0.0176	1.5394
	C	1.5729	0.1263	1.9652
	C	2.2843	-1.0265	-0.0938
	H	-0.4750	0.8604	1.9303
	H	0.3141	0.9759	-0.3918
	H	-1.0014	-0.1959	-0.3087
	H	1.9495	1.1315	1.7118
	H	1.6667	0.0325	3.0508
	H	-0.3164	-0.9109	1.9366
	C	2.9957	-1.8594	2.2659
	H	2.2503	-2.2792	2.9613
	H	3.7144	-1.3055	2.8927
	C	3.7364	-3.0117	1.5755
	H	4.6063	-3.3000	2.1760
	H	3.0806	-3.8824	1.4887
	C	4.1334	-2.6121	0.1617
	H	4.5964	-3.4576	-0.3562
	H	4.8773	-1.7944	0.1684
	C	0.6693	-1.2811	-1.9438
	H	-0.4055	-1.3858	-2.1315
	H	1.0013	-0.4039	-2.5326
	C	1.4043	-2.5325	-2.4189
	H	1.3442	-2.6134	-3.5100
	H	0.9237	-3.4112	-1.9777
	C	2.8628	-2.5019	-1.9649
	H	3.4276	-1.7488	-2.5450
	H	3.3369	-3.4685	-2.1639
	N	0.8508	-1.1001	-0.5078
	N	2.3733	-0.9141	1.3521
	N	2.9009	-2.2799	-0.5290
	C	3.0055	0.2065	-0.7162

Continue on the next page

H	2.6013	1.1431	-0.3285
H	4.0661	0.1723	-0.4553
H	2.9207	0.2430	-1.8040

I-1

G (gas phase) [Hartrees]			-595.856585	
G (SMD-w) [Hartrees]			-595.863740	
G (SMD-ace) [Hartrees]			-595.870494	
Num. Im. Freq.			0	
Opt Coord		x	y	z
	C	0.0409	0.016	0.0169
	C	0.0834	0.004	1.5436
	C	1.5447	0.033	2.0034
	C	2.2620	-1.062	-0.0667
	H	-0.4636	0.864	1.9472
	H	0.3705	1.006	-0.3575
	H	-0.9865	-0.120	-0.3385
	H	1.9795	1.009	1.7528
	H	1.6210	-0.068	3.0901
	H	-0.4051	-0.906	1.9070
	C	2.2288	-2.344	2.0014
	H	1.3566	-2.889	1.6148
	H	2.0947	-2.218	3.0798
	C	3.5194	-3.124	1.7054
	H	4.2954	-2.872	2.4360
	H	3.3242	-4.200	1.7816
	C	3.9963	-2.755	0.2844
	H	4.4437	-3.620	-0.2146
	H	4.7772	-1.978	0.3318
	C	0.6815	-1.264	-1.9463
	H	-0.3918	-1.326	-2.1602
	H	1.0625	-0.401	-2.5261

Continue on the next page

C	1.3786	-2.544	-2.4059
H	1.3393	-2.620	-3.4984
H	0.8515	-3.403	-1.9787
C	2.8277	-2.577	-1.9169
H	3.4398	-1.854	-2.4881
H	3.2628	-3.565	-2.1000
N	0.8394	-1.084	-0.5073
N	2.3596	-1.016	1.3975
N	2.8346	-2.345	-0.4860
C	3.0508	0.142	-0.6355
H	2.6527	1.094	-0.2816
H	4.0921	0.075	-0.3134
H	3.0258	0.165	-1.7262

TS2

G (gas phase) [Hartrees]			-595.849451	
G (SMD-w) [Hartrees]			-595.855858	
G (SMD-ace) [Hartrees]			-595.862864	
Num. Im. Freq.			0	
Opt Coord		x	y	z
	C	0.0502	-0.0909	0.0427
	C	0.1566	0.0376	1.5669
	C	1.6268	0.0938	2.0192
	C	2.3795	-0.8816	-0.0620
	H	-0.3769	0.9337	1.9034
	H	0.2243	0.8929	-0.4318
	H	-0.9701	-0.3836	-0.2317
	H	2.0591	1.0690	1.7794
	H	1.6961	-0.0181	3.1063
	H	-0.3390	-0.8207	2.0306
	C	2.0878	-2.2788	1.9443
	H	1.0246	-2.5040	1.7954

Continue on the next page

H	2.2633	-2.2210	3.0238
C	2.9362	-3.4046	1.3171
H	3.4653	-3.9744	2.0898
H	2.2866	-4.1036	0.7806
C	3.9255	-2.7918	0.3292
H	4.4357	-3.5709	-0.2441
H	4.6989	-2.2425	0.8828
C	0.7527	-1.3829	-1.8837
H	-0.3146	-1.5817	-2.0383
H	1.0033	-0.5098	-2.5200
C	1.5908	-2.5818	-2.3144
H	1.4647	-2.7660	-3.3881
H	1.2537	-3.4696	-1.7682
C	3.0588	-2.2969	-1.9881
H	3.4136	-1.5034	-2.6686
H	3.6826	-3.1719	-2.1900
N	0.9567	-1.1197	-0.4657
N	2.4509	-0.9529	1.4039
N	3.2552	-1.9105	-0.6065
C	2.9254	0.4818	-0.5526
H	2.2801	1.3348	-0.3369
H	3.8974	0.6475	-0.0843
H	3.0682	0.4471	-1.6346

I-2

G (gas phase) [Hartrees]			-595.850828	
G (SMD-w) [Hartrees]			-595.859388	
G (SMD-ace) [Hartrees]			-595.864909	
Num. Im. Freq.			0	
Opt Coord		x	y	z
	C	0.0000	0.0000	0.0000
	C	0.0000	0.0000	1.5326

Continue on the next page

C	1.4335	0.0000	2.0945
C	2.3304	-0.8043	-0.0067
H	-0.5543	0.8693	1.9040
H	0.2097	1.0188	-0.3762
H	-1.0010	-0.2567	-0.3670
H	1.8880	0.9874	1.9823
H	1.4226	-0.2135	3.1687
H	-0.5339	-0.8885	1.8833
C	1.8949	-2.3549	1.9085
H	0.8183	-2.4301	2.1089
H	2.4088	-2.5086	2.8667
C	2.2929	-3.4525	0.9244
H	2.4881	-4.3859	1.4646
H	1.4789	-3.6312	0.2171
C	3.5249	-2.9898	0.1604
H	3.8625	-3.7604	-0.5385
H	4.3470	-2.8207	0.8707
C	0.8434	-1.0506	-1.9982
H	-0.2043	-1.2440	-2.2586
H	1.1113	-0.0885	-2.4805
C	1.7468	-2.1529	-2.5403
H	1.7159	-2.1599	-3.6365
H	1.3900	-3.1267	-2.1867
C	3.1735	-1.9013	-2.0451
H	3.5496	-0.9865	-2.5245
H	3.8499	-2.7024	-2.3547
N	0.9371	-0.9856	-0.5434
N	2.2840	-1.0036	1.4453
N	3.2664	-1.7668	-0.5966
C	2.9156	0.5976	-0.3060
H	2.2465	1.4217	-0.0575
H	3.8398	0.7056	0.2630
H	3.1531	0.6871	-1.3666

TS3

G (gas phase) [Hartrees]		-595.848474		
G (SMD-w) [Hartrees]		-595.854228		
G (SMD-ace) [Hartrees]		-595.861993		
Num. Im. Freq.		1		
Opt Coord		x	y	z
	C	-0.0166	0.1605	0.0025
	C	-0.0932	-0.0896	1.5066
	C	1.3245	-0.1099	2.0845
	C	2.2801	-0.7909	-0.0578
	H	-0.6978	0.6852	1.9924
	H	0.2787	1.2117	-0.1857
	H	-1.0053	0.0347	-0.4540
	H	1.7533	0.8970	2.0377
	H	1.3180	-0.3943	3.1414
	H	-0.5812	-1.0542	1.6853
	C	1.9877	-2.4463	1.6938
	H	1.1746	-2.8855	1.0962
	H	1.7116	-2.5254	2.7497
	C	3.3097	-3.1509	1.4358
	H	4.0554	-2.7040	2.0993
	H	3.2516	-4.2201	1.6672
	C	3.7149	-2.9574	-0.0349
	H	3.4729	-3.8782	-0.5862
	H	4.8108	-2.8449	-0.1023
	C	0.8311	-0.7196	-2.0808
	H	-0.2217	-0.7797	-2.3815
	H	1.2077	0.2495	-2.4645
	C	1.6249	-1.8608	-2.7080
	H	1.6369	-1.7579	-3.7998
	H	1.1415	-2.8106	-2.4548
	C	3.0473	-1.8611	-2.1454
	H	3.6037	-0.9992	-2.5523

Continue on the next page

H	3.5888	-2.7511	-2.4784
N	0.8837	-0.8018	-0.6251
N	2.2142	-1.0346	1.3822
N	3.0230	-1.8637	-0.6982
C	3.0042	0.5570	-0.2955
H	2.4452	1.4171	0.0784
H	3.9685	0.5195	0.2143
H	3.1790	0.7257	-1.3592

2-staggered

G (gas phase) [Hartrees]		-595.859573		
G (SMD-w) [Hartrees]		-595.870562		
G (SMD-ace) [Hartrees]		-595.874476		
Num. Im. Freq.		0		
Opt Coord		x	y	z
	C	0.0041	-0.0043	0.0037
	C	0.0118	-0.0410	1.5297
	C	1.4565	0.0185	2.0389
	C	2.3109	-0.9192	-0.0518
	H	-0.5713	0.7975	1.9273
	H	0.2606	1.0160	-0.3430
	H	-1.0046	-0.2099	-0.3742
	H	1.8743	1.0121	1.8518
	H	1.4949	-0.1307	3.1227
	H	-0.4688	-0.9633	1.8716
	C	2.0349	-2.3209	1.9880
	H	1.0076	-2.6669	1.7832
	H	2.1394	-2.2330	3.0750
	C	3.0114	-3.3390	1.4310
	H	4.0355	-3.0541	1.6929
	H	2.8044	-4.3321	1.8445
	C	2.8494	-3.3505	-0.0769

Continue on the next page

H	1.8378	-3.7163	-0.3213
H	3.5661	-4.0365	-0.5418
C	0.8029	-1.0133	-2.0209
H	-0.2521	-1.1596	-2.2816
H	1.0941	-0.0359	-2.4538
C	1.6556	-2.1185	-2.6384
H	1.6601	-2.0222	-3.7302
H	1.2147	-3.0917	-2.3991
C	3.0829	-2.0377	-2.0850
H	3.5817	-1.1462	-2.4765
H	3.6796	-2.8932	-2.4173
N	0.9024	-1.0131	-0.5604
N	2.3142	-0.9990	1.4212
N	3.1186	-2.0160	-0.6184
C	3.0139	0.4038	-0.4339
H	2.4920	1.2749	-0.0396
H	4.0234	0.3806	-0.0227
H	3.0752	0.5400	-1.5129

**1**-eclipsed				
G (gas phase) [Hartrees]			-595.856579	
G (SMD-w) [Hartrees]			-595.870784	
G (SMD-ace) [Hartrees]			-595.873243	
Num. Im. Freq.			1	
Opt Coord		x	y	z
	C	-0.0001	-0.0001	-0.0000
	C	-0.0000	0.0001	1.5218
	C	1.4362	0.0002	2.0250
	C	2.1988	-1.1033	-0.0368
	H	-0.5385	0.8744	1.9035
	H	0.3565	0.9805	-0.3747
	H	-1.0189	-0.1242	-0.3825

Continue on the next page

H	1.9081	0.9805	1.8111
H	1.4603	-0.1225	3.1131
H	-0.5022	-0.9042	1.8784
C	3.4797	-1.2832	2.0561
H	3.3344	-1.3025	3.1415
H	4.1618	-0.4342	1.8477
C	4.1158	-2.5819	1.5819
H	5.1291	-2.6808	1.9866
H	3.5131	-3.4238	1.9358
C	4.1479	-2.6008	0.0604
H	4.4888	-3.5762	-0.3028
H	4.8823	-1.8564	-0.3081
C	0.6498	-1.2808	-1.9395
H	-0.4221	-1.2983	-2.1634
H	1.0744	-0.4316	-2.5122
C	1.3066	-2.5801	-2.3829
H	1.2610	-2.6785	-3.4731
H	0.7716	-3.4215	-1.9322
C	2.7525	-2.6009	-1.9084
H	3.3448	-1.8575	-2.4792
H	3.2070	-3.5770	-2.1093
N	0.7924	-1.1171	-0.4983
N	2.1680	-1.1175	1.4428
N	2.8078	-2.3814	-0.4686
C	2.9860	0.1384	-0.5945
H	3.3741	0.7684	0.2087
H	3.8404	-0.1619	-1.2049
H	2.3547	0.7729	-1.2203

2-eclipsed

G (gas phase) [Hartrees]	-595.856468
G (SMD-w) [Hartrees]	-595.867366

Continue on the next page

G (SMD-ace) [Hartrees]		-595.871162		
Num. Im. Freq.		1		
Opt Coord		x	y	z
	C	0.0002	0.0005	0.0002
	C	-0.0000	-0.0001	1.5252
	C	1.4484	-0.0002	2.0226
	C	2.2531	-1.0443	-0.0280
	H	-0.5356	0.8790	1.9012
	H	0.3258	0.9955	-0.3650
	H	-1.0162	-0.1481	-0.3831
	H	1.9111	0.9602	1.7717
	H	1.4943	-0.0942	3.1123
	H	-0.5289	-0.8863	1.8905
	C	1.8812	-2.3741	2.0450
	H	0.8349	-2.6599	1.8453
	H	1.9887	-2.2650	3.1300
	C	2.7933	-3.4654	1.5161
	H	3.8326	-3.2391	1.7744
	H	2.5236	-4.4328	1.9537
	C	2.6340	-3.5059	0.0075
	H	1.6023	-3.8141	-0.2310
	H	3.3075	-4.2466	-0.4379
	C	0.7358	-1.1062	-1.9929
	H	-0.3270	-1.1849	-2.2507
	H	1.0956	-0.1627	-2.4510
	C	1.5143	-2.2792	-2.5789
	H	1.5213	-2.2164	-3.6731
	H	1.0202	-3.2179	-2.3082
	C	2.9439	-2.2512	-2.0303
	H	3.4641	-1.3770	-2.4360
	H	3.5109	-3.1302	-2.3535
	N	0.8375	-1.0741	-0.5336
	N	2.2460	-1.0880	1.4463

Continue on the next page

N	2.9882	-2.2051	-0.5648
C	3.0361	0.2372	-0.4511
H	3.3882	0.7687	0.4315
H	3.9226	-0.0370	-1.0206
H	2.4391	0.9264	-1.0534

S4 Topological analysis of electron density

This section presents the results obtained from the topological analysis of the theoretical electron density. The tables provide selected topological properties of the electron density at the non-covalent interactions' bond critical points (BPC). These properties include the *electron density*, $\rho(\mathbf{r})$; the *Laplacian* of the *electron density*, $\nabla^2\rho(\mathbf{r})$; and the *kinetic*, $G(\mathbf{r})$, and *potential*, $V(\mathbf{r})$, *energy densities*. All of them are reported in atomic units. Additionally, the tables present the values of two energy indicators introduced by Espinosa et al. [12]: $|V(\mathbf{r})|/G(\mathbf{r})$ and $H(\mathbf{r})/\rho(\mathbf{r})$, where $H(\mathbf{r})$ is the energy density written as $H(\mathbf{r}) = G(\mathbf{r}) + V(\mathbf{r})$. The latter indicator is referred to as *bond degree* (BD).

Table S15: Topological properties of electron density at BCPs: analysis of bond paths of Figure 6.

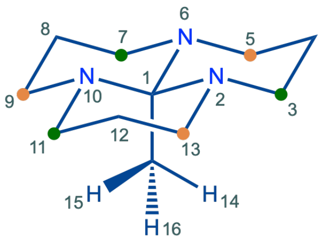

BCP	Bond	$\rho(\mathbf{r})$	$\nabla^2\rho(\mathbf{r})$	$G(\mathbf{r})$	$V(\mathbf{r})$	$\|V(\mathbf{r})\|/G(\mathbf{r})$	$H(\mathbf{r})/\rho(\mathbf{r})$
Alternated conformer							
40	H14 ··· H3	0.0105	0.0435	0.0087	-0.0065	0.7497	0.2072
41	H14 ··· H5	0.0104	0.0427	0.0085	-0.0064	0.7482	0.2076
39	H15 ··· H13	0.0111	0.0451	0.0091	-0.0069	0.7615	0.1955
38	H16 ··· H7	0.0112	0.0456	0.0092	-0.0070	0.7624	0.1953
42	H16 ··· H9	0.0102	0.0434	0.0086	-0.0064	0.7410	0.2183

Continue on the next page

Table S15: (Continued.)

Eclipsed conformer							
39	H14 ··· H3	0.0120	0.0488	0.0099	-0.0077	0.7726	0.1884
40	H15 ··· H11	0.0120	0.0488	0.0099	-0.0077	0.7726	0.1884
38	H16 ··· H7	0.0120	0.0488	0.0099	-0.0077	0.7726	0.1884

Table S16: Topological properties of electron density at BCPs. Analysis of bond paths of Figure 10 (top).

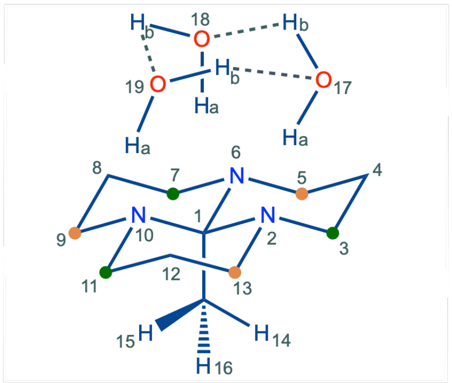

BCP	Bond	$\rho(\mathbf{r})$	$\nabla^2\rho(\mathbf{r})$	$G(\mathbf{r})$	$V(\mathbf{r})$	$\lvert V(\mathbf{r})\rvert/G(\mathbf{r})$	$H(\mathbf{r})/\rho(\mathbf{r})$
Type interaction N ··· H							
46	N2 ··· H17a	0.0346	0.0886	0.0253	-0.0285	1.1249	-0.0914
44	N6 ··· H18a	0.0346	0.0886	0.0253	-0.0285	1.1249	-0.0914
45	N10 ··· H19a	0.0346	0.0886	0.0253	-0.0285	1.1249	-0.0914
Type interaction O ··· H							
50	O17 ··· H19b	0.0017	0.0070	0.0012	-0.0007	0.5828	0.3069
52	O18 ··· H17b	0.0017	0.0070	0.0012	-0.0007	0.5828	0.3069
51	O19 ··· H18b	0.0017	0.0070	0.0012	-0.0007	0.5828	0.3069
Type interaction H ··· H							
49	H14 ··· H3	0.0121	0.0490	0.0100	-0.0077	0.7733	0.1879
47	H15 ··· H11	0.0121	0.0490	0.0100	-0.0077	0.7733	0.1879
48	H16 ··· H7	0.0121	0.0490	0.0100	-0.0077	0.7733	0.1879

Table S17: Topological properties of electron density at BCPs. Analysis of bond paths of Figure 10 (bottom) and Figure 11.

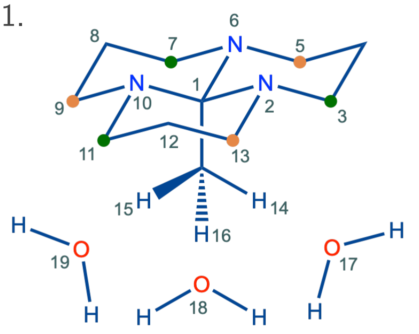

BCP	Bond	$\rho(\mathbf{r})$	$\nabla^2\rho(\mathbf{r})$	$G(\mathbf{r})$	$V(\mathbf{r})$	$\lvert V(\mathbf{r})\rvert/G(\mathbf{r})$	$H(\mathbf{r})/\rho(\mathbf{r})$
			**Eclipsed** tricyclic orthoamide, Figure 10 (bottom)				
Water molecule 1 (H_2O17)							
46	H14 ··· H3	0.0119	0.0495	0.0100	-0.0077	0.7653	0.1981
47	O17 ··· H14	0.0083	0.0267	0.0054	-0.0042	0.7693	0.1510
51	O17 ··· H3	0.0062	0.0199	0.0039	-0.0029	0.7310	0.1695
53	O17 ··· H13	0.0055	0.0183	0.0036	-0.0025	0.7103	0.1857
Water molecule 2 (H_2O18)							
44	H16 ··· H7	0.0119	0.0495	0.0100	-0.0077	0.7653	0.1981
49	O18 ··· H16	0.0083	0.0267	0.0054	-0.0042	0.7693	0.1509
54	O18 ··· H5	0.0055	0.0183	0.0036	-0.0025	0.7103	0.1857
52	O18 ··· H7	0.0062	0.0199	0.0039	-0.0029	0.7310	0.1695
Water molecule 3 (H_2O19)							
45	H15 ··· H11	0.0119	0.0495	0.0100	-0.0077	0.7653	0.1981
48	O19 ··· H15	0.0083	0.0267	0.0054	-0.0042	0.7694	0.1509
55	O19 ··· H9	0.0055	0.0183	0.0036	-0.0025	0.7103	0.1857
50	O19 ··· H11	0.0062	0.0199	0.0039	-0.0029	0.7310	0.1695
			**Alternate** tricyclic orthoamide, Figure 11				
Water molecule 1 (H_2O17)							
45	H14 ··· H13	0.0125	0.0498	0.0102	-0.0080	0.7836	0.1770
55	O17 ··· H14	0.0044	0.0150	0.0028	-0.0019	0.6823	0.2031
52	O17 ··· H3	0.0054	0.0179	0.0035	-0.0025	0.7074	0.1874
47	O17 ··· H13	0.0061	0.0198	0.0039	-0.0028	0.7253	0.1751
Water molecule 2 (H_2O18)							

Continue on the next page

Table S17: (Continued.)

44	H16 ··· H5	0.0125	0.0498	0.0102	-0.0080	0.7836	0.1770
53	O18 ··· H16	0.0044	0.0150	0.0028	-0.0019	0.6820	0.2033
49	O18 ··· H5	0.0061	0.0198	0.0039	-0.0028	0.7255	0.1750
50	O18 ··· H7	0.0054	0.0179	0.0035	-0.0025	0.7074	0.1874
Water molecule 3 (H_2O19)							
46	H15 ··· H9	0.0125	0.0498	0.0102	-0.0080	0.7836	0.1770
54	O19 ··· H15	0.0044	0.0150	0.0028	-0.0019	0.6820	0.2033
48	O19 ··· H9	0.0061	0.0198	0.0039	-0.0028	0.7255	0.1750
51	O19 ··· H11	0.0054	0.0179	0.0035	-0.0025	0.7074	0.1874

Table S18: Topological properties of electron density at BCPs. Analysis of bond paths of Figure 9.

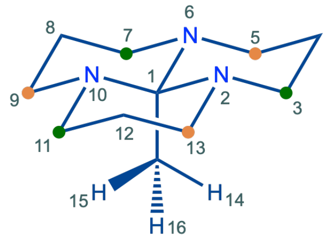

BCP	Bond	$\rho(\mathbf{r})$	$\nabla^2\rho(\mathbf{r})$	$G(\mathbf{r})$	$V(\mathbf{r})$	$\|V(\mathbf{r})\|/G(\mathbf{r})$	$H(\mathbf{r})/\rho(\mathbf{r})$
Type interaction H ··· H							
150	H14 ··· H	0.0115	0.0453	0.0092	-0.0072	0.7739	0.1813
161	H14 ··· H	0.0105	0.0429	0.0086	-0.0065	0.7506	0.2050
179	H14 ··· H	0.0050	0.0151	0.0029	-0.0021	0.7171	0.1650
188	H14 ··· H	0.0035	0.0112	0.0021	-0.0014	0.6667	0.1989
190	H14 ··· H	0.0030	0.0090	0.0017	-0.0011	0.6629	0.1867
160	H15 ··· H	0.0105	0.0438	0.0088	-0.0066	0.7487	0.2090
164	H15 ··· H	0.0104	0.0424	0.0085	-0.0064	0.7501	0.2045
154	H16 ··· H	0.0111	0.0449	0.0091	-0.0069	0.7628	0.1936
Type interaction N ··· H							
134	N2 ··· H	0.0037	0.0100	0.0019	-0.0013	0.6974	0.1575
136	N2 ··· H	0.0024	0.0074	0.0014	-0.0009	0.6355	0.2062
128	N6 ··· H	0.0101	0.0307	0.0065	-0.0053	0.8126	0.1204
113	N10 ··· H	0.0145	0.0391	0.0090	-0.0082	0.9124	0.0544

S4 .1 Interaction energy calculation

We calculated the pairwise intermolecular interaction energy using the energy method implemented in CrystalExplorer [13]. This method expresses the total energy of interaction (E_{tot}) between nearest-neighbor molecular pairs as the sum of four components: electrostatic (E_{ele}), polarization (E_{pol}), dispersion (E_{dis}), and repulsion (E_{rep}) (equation 1).

$$E_{\text{tot}} = \text{k}_{\text{ele}}\, E_{\text{ele}} + \text{k}_{\text{pol}}\, E_{\text{pol}} + \text{k}_{\text{dis}}\, E_{\text{dis}} + \text{k}_{\text{rep}}\, E_{\text{rep}} \tag{1}$$

The program provides scale factors represented by the k coefficients. The interaction energies for the ATO and HTO systems, considering all molecules within a radius of 5.0 Å, are presented in the following tables.

Table S19: Interaction energy analysis of the ATO system calculated with the B3LYP/6-31G(d,p) approximation. The energies (E) are in kJ/mol and the radial distance (R) in Å. The color-coded interaction mapping corresponds to a molecular cluster of 5 Å.

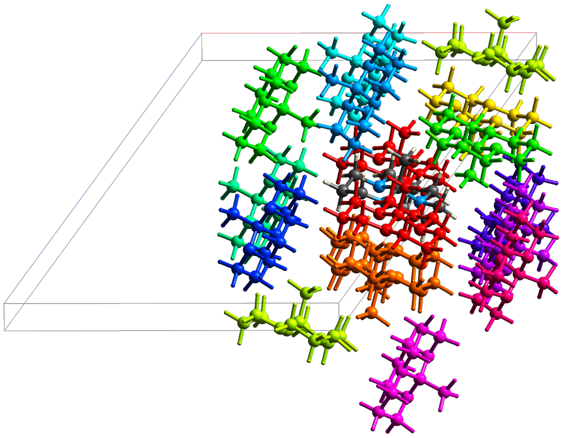

	N	Symop	R	E_{ele}	E_{pol}	E_{dis}	E_{rep}	E_{tot}
	2	x, y, z	8.54	-2.4	-0.1	-15.0	10.3	-9.3
	2	−x,y+1/2,−z+1/2	5.87	-15.4	-7.0	-42.1	33.7	-37.3
	1	−x, −y, −z	6.92	-7.8	-0.1	-34.2	29.2	-20.0
	2	x,−y+1/2,z+1/2	8.84	-0.2	-0.1	-1.7	0.0	-1.8

Continue on the next page

Table S19: (Continued.)

	1	−x, −y, −z	6.81	-2.5	-0.1	-23.0	10.9	-15.9
	1	–	7.93	0.0	0.0	0.0	0.0	0.0
	1	–	7.88	0.0	0.0	-0.1	0.0	-0.2
	1	–	7.71	0.0	0.0	-0.1	0.0	-0.2
	1	–	7.10	0.0	0.0	-0.1	0.0	-0.2
	1	–	8.34	0.0	0.0	-0.2	0.0	-0.1
	1	–	10.33	0.0	0.0	-0.0	0.0	-0.0
	1	–	6.42	0.0	0.0	-0.1	0.0	-0.1
	1	–	9.79	0.0	0.0	-0.0	0.0	-0.1
	1	–	11.03	0.0	0.0	-0.0	0.0	0.0

Scale factors for benchmarked energy model

Energy model	k_{ele}	k_{pol}	k_{dis}	k_{rep}
CE-HF ... HF/3-21G electron densities	1.019	0.651	0.901	0.811
CE-B3LYP ... B3LYP/6-31G(d,p) electron densities	1.057	0.740	0.871	0.618

Table S20: Interaction energy analysis of the HTO system calculated with the B3LYP/6-31G(d,p) approximation. The energies (E) are in kJ/mol and the radial distance (R) in Å. The color-coded interaction mapping corresponds to a molecular cluster of 5 Å.

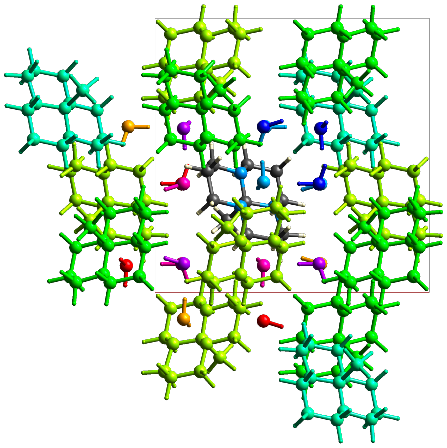

	N	Symop	R	E_{ele}	E_{pol}	E_{dis}	E_{rep}	E_{tot}
	3	–	7.00	-0.4	-0.1	-0.5	0.0	-0.9
	3	–	7.64	0.4	-0.1	-0.5	0.0	-0.1
	6	x+1/2,y,−z+1/2	7.27	-4.3	-0.2	-23.5	16.1	-15.2

Continue on the next page

Table S20: (Continued.)

6	x+1/2,−y+1/2,−z	8.52	-0.8	-0.1	-9.0	3.0	-6.9
3	−x, −y, −z	11.20	-0.0	-0.0	-1.2	0.0	-1.1
3	–	3.79	-47.9	-12.0	-16.7	52.7	-41.5
3	–	5.15	-2.3	-0.6	-2.6	0.5	-4.9
3	–	5.76	-0.3	-0.6	-3.7	1.1	-3.3
3	–	4.73	-4.6	-3.3	-6.8	9.5	-7.3

Scale factors for benchmarked energy model

Energy model	k_{ele}	k_{pol}	k_{dis}	k_{rep}
CE-HF ... HF/3-21G electron densities	1.019	0.651	0.901	0.811
CE-B3LYP ... B3LYP/6-31G(d,p) electron densities	1.057	0.740	0.871	0.618

References

[1] P. Seiler and J. D. Dunitz. An Eclipsed C(sp^3)–CH_3 Bond in a Crystalline Hydrated Tricyclic Orthoamide: Evidence for C–H· · · O hydrogen bonds. *Helv. Chim. Acta*, 26(11):1175–1177, 1989.

[2] M. J. Frisch, G. W. Trucks, H. B. Schlegel, G. E. Scuseria, M. A. Robb, J. R. Cheeseman, G. Scalmani, V. Barone, G. A. Petersson, H. Nakatsuji, X. Li, M. Caricato, A. V. Marenich, J. Bloino, B. G. Janesko, R. Gomperts, B. Mennucci, H. P. Hratchian, J. V. Ortiz, A. F. Izmaylov, J. L. Sonnenberg, D. Williams-Young, F. Ding, F. Lipparini, F. Egidi, J. Goings, B. Peng, A. Petrone, T. Henderson, D. Ranasinghe, V. G. Zakrzewski, J. Gao, N. Rega, G. Zheng, W. Liang, M. Hada, M. Ehara, K. Toyota, R. Fukuda, J. Hasegawa, M. Ishida, T. Nakajima, Y. Honda, O. Kitao, H. Nakai, T. Vreven, K. Throssell, J. A. Montgomery, Jr., J. E. Peralta, F. Ogliaro, M. J. Bearpark, J. J. Heyd, E. N. Brothers, K. N. Kudin, V. N. Staroverov, T. A. Keith, R. Kobayashi, J. Normand, K. Raghavachari, A. P. Rendell, J. C. Burant, S. S. Iyengar, J. Tomasi, M. Cossi, J. M. Millam, M. Klene, C. Adamo, R. Cammi, J. W. Ochterski, R. L. Martin, K. Morokuma, O. Farkas, J. B. Foresman, and D. J. Fox. Gaussian˜16 Revision C.01, 2016. Gaussian Inc. Wallingford CT.

[3] A. D. Becke. Density-functional thermochemistry. iii. the role of exact exchange. *J. Chem. Phys.*, 98:5648–5652, 1993.

[4] C. Lee, W. Yang, and R. G. Parr. Development of the Colle-Salvetti correlation-energy formula into a functional of the electron density. *Phys. Rev. B*, 37:785–789, 1988.

[5] S. H. Vosko, L. Wilk, and M. Nusair. Accurate spin-dependent electron liquid correlation energies for local spin density calculations: a critical analysis. *Can. J. Phys.*, 58:1200–1211, 1980.

[6] P. J. Stephens, F. J. Devlin, C. F. Chabalowski, and M. J. Frisch. Ab Initio Calculation of Vibrational Absorption and Circular Dichroism Spectra Using Density Functional Force Fields. *J. Phys. Chem.*, 98:11623–11627, 1994.

[7] R. Ditchfield, W. J. Hehre, and J. A. Pople. Self-Consistent Molecular-Orbital Methods. IX. An Extended Gaussian-Type Basis for Molecular-Orbital Studies of Organic Molecules. *J. Chem. Phys.*, 54:724–728, 1971.

[8] P. C. Hariharan and J. A. Pople. The influence of polarization functions on molecular orbital hydrogenation energies. *Theor. Chim. Acta*, 28:213–222, 1973.

[9] S. Grimme, S. Ehrlich, and L. Goerigk. Effect of the damping function in dispersion corrected density functional theory. *J. Comput. Chem.*, 32, 2011.

[10] A. V. Marenich, C. J. Cramer, and D. G. Truhlar. Universal Solvation Model Based on Solute Electron Density and on a Continuum Model of the Solvent Defined by the Bulk Dielectric Constant and Atomic Surface Tensions. *J. Phys. Chem. B*, 113(18):6378–6396, 2009.

[11] G. R. Weisman, V. Johnson, and R. E. Fiala. Tricyclic orthoamides: Effects of lone-pair orientation upon NMR spectra. *Tetrahedron Lett.*, 21(38):3635–3638, 1980.

[12] E. Espinosa, I. Alkorta, J. Elguero, and E. Molins. From weak to strong interactions: A comprehensive analysis of the topological and energetic properties of the electron density distribution involving X–H$\cdots$F–Y systems. *J. Chem. Phys.*, 117(12):5529–5542, 2002.

[13] P. R. Spackman, M. J. Turner, J. J. McKinnon, S. K. Wolff, D. J. Grimwood, D. Jayatilaka, and M. A. Spackman. *CrystalExplorer*: a program for Hirshfeld surface analysis, visualization and quantitative analysis of molecular crystals. *J. Appl. Crystallogr.*, 54(3), 2021.
